# Gene Expression Signature-Based Screening Identifies New Broadly Effective Influenza A Antivirals

**DOI:** 10.1371/journal.pone.0013169

**Published:** 2010-10-04

**Authors:** Laurence Josset, Julien Textoris, Béatrice Loriod, Olivier Ferraris, Vincent Moules, Bruno Lina, Catherine N'Guyen, Jean-Jacques Diaz, Manuel Rosa-Calatrava

**Affiliations:** 1 Centre National de la Recherche Scientifique (CNRS) FRE 3011 Virologie et Pathologie Humaine, Université Lyon 1, Lyon, France; 2 Laboratoire de Virologie Centre de Biologie et de Pathologie Est, Hospices Civils de Lyon, Lyon, France; 3 Institut National de la Santé et de la Recherche Médicale (INSERM) U928 Technologies Avancées pour le Génome et la Clinique, Université de la Méditerranée, Marseille, France; 4 Centre National de la Recherche Scientifique (CNRS) UMR 5534, Centre Léon Bérard, Centre de Génétique Moléculaire et Cellulaire, Université Lyon 1, Lyon, France; 5 Service d'anesthésie et de réanimation Hôpital Nord, Assistance Publique - Hôpitaux de Marseille, Marseille, France; Hallym University, Republic of Korea

## Abstract

Classical antiviral therapies target viral proteins and are consequently subject to resistance. To counteract this limitation, alternative strategies have been developed that target cellular factors. We hypothesized that such an approach could also be useful to identify broad-spectrum antivirals. The influenza A virus was used as a model for its viral diversity and because of the need to develop therapies against unpredictable viruses as recently underlined by the H1N1 pandemic. We proposed to identify a gene-expression signature associated with infection by different influenza A virus subtypes which would allow the identification of potential antiviral drugs with a broad anti-influenza spectrum of activity. We analyzed the cellular gene expression response to infection with five different human and avian influenza A virus strains and identified 300 genes as differentially expressed between infected and non-infected samples. The most 20 dysregulated genes were used to screen the connectivity map, a database of drug-associated gene expression profiles. Candidate antivirals were then identified by their inverse correlation to the query signature. We hypothesized that such molecules would induce an unfavorable cellular environment for influenza virus replication. Eight potential antivirals including ribavirin were identified and their effects were tested in vitro on five influenza A strains. Six of the molecules inhibited influenza viral growth. The new pandemic H1N1 virus, which was not used to define the gene expression signature of infection, was inhibited by five out of the eight identified molecules, demonstrating that this strategy could contribute to identifying new broad anti-influenza agents acting on cellular gene expression. The identified infection signature genes, the expression of which are modified upon infection, could encode cellular proteins involved in the viral life cycle. This is the first study showing that gene expression-based screening can be used to identify antivirals. Such an approach could accelerate drug discovery and be extended to other pathogens.

## Introduction

Antiviral drug development is currently based on two approaches: i) the conventional approach of inhibiting the activity of a viral enzyme which often leads to the emergence of drug resistant viruses due to viral genomic variability and ii) the more recent approach of targeting cellular factors that are required for viral replication. Indeed, coding for a limited number of proteins, viruses hijack the cellular machinery and rely on many host proteins for their replication. The major recognized advantage of targeting a host factor is therefore to limit the development of resistance as the virus cannot replace a missing cellular protein [1]. Such an approach has been used in antiretroviral therapy with the development of a CCR5 antagonist showing promise as an anti-HIV drug [2]. We have also demonstrated that this strategy is efficient at inhibiting the replication of herpes viruses resistant to conventional antivirals [3]. In influenza research, the effective in vitro and in vivo inhibition of two different cellular pathways without inducing resistance has been reported, and both are currently undergoing preclinical trials (recently reviewed in [4]).

Targeting cellular proteins may provide another crucial advantage: if a cellular pathway is critical to the viral cycle, agents that target such a pathway should represent potential broad-spectrum antivirals. The influenza virus represents a constant threat to public health due to the emergence of new viral strains and is therefore an ideal model on which to test this hypothesis.

Belonging to the orthomyxoviridae family, influenza viruses have genomes composed of single-stranded RNA and are classified into three types: A, B and C according to their internal protein sequences [5]. The influenza A viruses are further subtyped based on the antigenicity of the two envelope glycoproteins hemagglutinin (HA) [H1 to H16] and neuraminidase (NA) [N1 to N9]. All influenza A subtypes are endemic in aquatic birds but only two, H1N1 and H3N2, are presently circulating among humans. Since the influenza genome is segmented, two different viral strains infecting the same cell are able to reassort their genomic segments. Variability can also be due to the low fidelity of the viral RNA polymerase, which causes yearly epidemics owing to an antigenic drift in glycoproteins. Novel pathogenic strains of the influenza virus have also emerged with antigenically different HA and/or NA and have caused three pandemics in the 20th century: the Spanish influenza (H1N1) in 1918, responsible for approximately 50 million deaths; the Asian influenza (H2N2) in 1957 during which about 2–4 million people died; and the Hong Kong influenza (H3N2) in 1968 responsible for 1–2 million deaths [6].

Considering this pandemic potential and with up to 500,000 annual deaths worldwide during usual winter outbreaks, influenza A viruses represent a major public health concern [7]. Prevention relies on vaccination which has several major limitations including the lag time for vaccine preparation and the low vaccination coverage rate. Once a patient becomes infected, the current etiologic treatment of flu relies on M2 channel blockers or NA inhibitors [8]. However, these existing therapies are inappropriate for use in cases of severe infection and may be limited due to the risk of rapid emergence of drug resistant viruses. Thus there is an obvious need to complement existing therapies with new anti-influenza drugs.

To search for new antivirals, we hypothesized that common viral effects on cell metabolism should occur after infection with different avian and human influenza viruses and that this pattern should lead to the identification of drugs effective on all influenza A viruses potentially. We first sought to identify a common gene expression signature following the infection with different human and avian influenza A viruses. While several microarray analyses have already compared the pandemic 1918 H1N1 virus [9], [10] or some H5N1 strain [11], [12] to other less pathogenic strains, our study is the first to demonstrate that a global influenza-induced gene-expression signature can be defined. This proof-of-concept study was conducted on a home-made nylon array using a human pulmonary epithelial cell line infected by five influenza A virus subtypes (H1N1, H3N2, H5N1, H5N2 and H7N1). Using this signature, we determined if molecules disturbing this pattern of infection would have a broad-influenza antiviral effect. By consulting the Connectivity Map, a database of drug-associated gene expression profiles [13], [14], we identified molecules that induced gene expression changes after cell treatment that were mainly opposite to those induced by infection. These molecules were tested in vitro for their effect on the five different viruses. To confirm our methodology, we took the opportunity of using the new emerging pandemic H1N1 virus as a model to test the effect of these molecules on a new unknown virus.

## Materials and Methods

### 1 Cell lines and viruses

Cells of the human lung epithelial cell line A549 were grown as monolayers in Dulbecco's modified Eagle's medium (DMEM) supplemented with 10% fetal bovine serum, 2 mM L-glutamine, 100 U of penicillin/mL, and 100 µg of streptomycin sulfate/mL at 37°C.

Influenza viruses A/New Caledonia/20/99 (H1N1), A/Moscow/10/99 (H3N2), A/Lyon/969/09 (H1N1 SOIV), A/Turkey/582/2006 (H5N1), A/Finch/England/2051/94 (H5N2), and A/Chicken/Italy/2076/99 (H7N1) were produced in MDCK cells in EMEM supplemented with 2 mM L-glutamine, 100U of penicillin/mL, 100 µg of streptomycin sulfate/mL and 1 µg of trypsin/mL. Viruses were titrated to determine tissue culture infection dose 50% (TCID50) in MDCK cells as described in our previous study [15].

For the microarray analysis, A549 cells were infected for 24 h at 37°C with influenza viruses at a multiplicity of infection (moi) of 1 in DMEM supplemented with 2 mM L-glutamine, 100 U of penicillin/mL, 100 µg of streptomycin sulfate/mL and 0.5 µg of trypsin/mL (infection medium). This moi was chosen to ensure that 100% of the cells were infected 24 h postinfection. The microarray experiments were performed in five independent replicates.

For kinetics on A549 cells, confluent cells were infected with influenza viruses at a moi of 0.1 or 2 for one hour under a minimal volume of infection medium at 37°C. The cells were then overlaid with fresh infection medium and incubated at 37°C. Samples of supernatants were collected at defined time points and stored at −80°C until end point titration assays (TCID50) in MDCK cells.

### 2 RNA preparation and hybridization to the gene chip

Total RNA was extracted from cell pellets using an RNeasy Mini Kit (Qiagen,Valencia,CA) for the BSL2 viruses. For H5N1 infections, total RNA was extracted with Trizol LS (Invitrogen). mRNAs were labeled with ^33^P for the reverse transcription using the Superscript III RT (Invitrogen), (α33P)dCTP and an oligodT25. Generated cDNAs were hybridized on home-made Nylon microarrays (HuSG9k) containing 9216 spotted IMAGE human cDNA clones, representing 8682 genes and 434 control clones [16]. Further details on the HuSG9k microarray are available on the TAGC website (http://tagc.univ-mrs.fr/). All membranes used in this study belonged to the same batch. After hybridization and exposure on Micro Imager, arrays were scanned in a Fuji BAS 5000 machine and hybridization signals quantified using the BZ Scan Software [17]. Primary data, in accordance with the proposed MIAME standards, are accessible through GEO Series accession number GSE22319 (http://www.ncbi.nlm.nih.gov/geo/query/acc.cgi?acc=GSE22319).

### 3 Data normalization and analysis

Data files were loaded and analyzed with R (v2.9.2) and Bioconductor (v2.4.1) [18], using the NylonArray library developed by the TAGC to support BZScan2 files (library available upon request). Raw data were normalized by quantile normalization. Supervised analysis (supervised methods aim at finding a set of genes whose expression profiles best correlate with a known phenotype) between groups *Infected* and *Mock* samples was conducted using the Significance Analysis of Microarray algorithm (SAM) [19], using the siggenes library (v1.18.0) [20]. All statistical analyses involved corrections for multiple comparisons (Benjamini and Hochberg) [21]. Agglomerative hierarchical clustering was performed by the pairwise average-linkage method using the Pearson correlation distance (Cluster 3.0, Eisen, Stanford University).

### 4 Quantitative real-time RT-PCR validation

To validate the microarray results with real-time RT-PCR assay, another set of A549 cells were infected with influenza viruses at a moi of 1 and total cell RNA was extracted at 24 hpi with Trizol LS (Invitrogen). Five hundred ng of total RNA were reverse transcribed using oligo(dT)18 and RevertAid M-MuLV (Fermentas) according to the manufacturer's instructions. One µL of cDNA was then amplified and analyzed in the 7500 Real Time PCR System (Applied Biosystems) using the Platinum(R) SYBR(R) Green qPCR SuperMix-UDG kit (Invitrogen) according to the manufacturer's instructions. Six genes were chosen according to their level of expression (Fold change in log_2_ > 2 or <−2) and the availability of primers for the quantitative PCR (Table S1). Glyceraldehyde 3-phosphate dehydrogenase (*GAPDH*) mRNA was used as an internal control. The reaction mix contained a total volume of 20 µL and the thermal cycling consisted of UDG incubation at 50°C for 2 min, 40 cycles of 95°C for 15 s and 60°C for 33 s for amplification. All data were normalized to the internal standard *GAPDH* mRNA. For each single-well amplification reaction, a threshold cycle (Ct) was observed in the exponential phase of amplification. Relative changes in gene expression were determined using the 2^ΔΔCt^ method as previously described [22] and reported as the n-fold difference relative to a control cDNA (mock cells) prepared in parallel with the experimental cDNAs (infected cells). Statistical significance was calculated using Welch's two sample t-test between mock and infected samples using R software.

### 5 *In silico* experiment: query the Connectivity Map with the infection signature

To select potential antivirals, an unbiased in silico search for molecules that reverse the infection signature identified in the present study was performed using the publicly available Connectivity Map database (build 02) [13]. The Connectivity Map (also known as CMAP) is a collection of genome-wide transcriptional data from cultured human cells treated with different kinds of molecules. The 20 most differentially expressed genes in the infection state (Fold Change in log_2_ > 2 or <−2) were selected from the initial 300 gene set identified by SAM. These were then mapped to the U133A probe sets in order to query the Connectivity Map database. In total, 28 U133A probe sets mapped to the selected genes from this study. The connectivity scores and p-values were obtained using the CMAP algorithm [13].

### 6 Molecules

2-aminobenzenesulfonamide (Sigma), calcium folinate (Sigma), harmol hydrochloride (MP Biomedical), merbromine (Sigma), midodrine (Sigma) and ribavirin (Valeant Pharmaceuticals) were dissolved in sterile water to a stock concentration of 5 g/L, 5 g/L, 4 g/L, 3.4 g/L, 5 g/L and 10 mM respectively. Rilmenidine (Sigma) was dissolved in dimethylsulfoxide (DMSO) to a stock concentration of 13 g/L and brinzolamide was in suspension at 10 g/L in the collyrium AZOPT.

Sulfameter (Sigma), pyrvinium (Sigma), moxalactam (Sigma) and methylbenzethoniumchloride (Sigma) were dissolved in sterile water to a stock concentration of 50 g/L. Alvespimycin (Sigma) was dissolved in sterile water to a concentration of 0.03 g/L. Sulodictil (Sigma) and DL-Thiorphan (Sigma) were dissolved in DMSO to a concentration of 50 g/L.

### 7 Viability assays

Cell viability was measured by the neutral red assay, an indicator of cytotoxicity used in cultures of different cell lines [23] with the same sensitivity as the MTT assay [24], [25]. The neutral red assay is based on the initial protocol described by Borenfreund and Puerner (1984) and determines the accumulation of the neutral red dye in the lysosomes of viable, uninjured cells. Cells were seeded into 96-well plates and treated with molecules or solvent. 72 h after treatment, cells were incubated for 3 h with neutral red dye (100 µg/ml) dissolved in serum free medium (DMEM). Cells were then washed with phosphate buffered saline (PBS) and fixed in a formol/calcium mix (40%/10%) for 1 min before being lysed with EtOH/AcCOOH, (50%/1%) followed by gentle shaking for 15 min until complete dissolution was achieved. Absorbance at 550 nm was measured using a microplate spectrophotometer system (Microplate Reader 2001, BioWhittaker) and results were presented as a ratio of control values.

### 8 Neuraminidase assay

Standard fluorometric endpoint assays used to monitor NA activity was recently shown to be suitable to quantify influenza virus in a high-throughput screening test [26]. Briefly, cell supernatants (25 µl) were transferred to a black 96-well plate and 75 µl of 2′-(4-methylumbelliferyl)-alpha-*N*-acetylneuraminic acid (MUNANA, Sigma Chemical Co.) to a final concentration of 50 µM were added. After incubation of the plate at 37°C for 1 hr, 150 µl stop solution (0.05 M glycine, pH 10.4) was added to each well and the fluorescence read on a FluoStar Opima (BMG Labtech) with excitation and emission filters of 355 nm and 460 nm respectively. Relative fluorescence units (RFU) were corrected by subtracting specific blanks, ie medium with or without molecules.

For the NA activity test on L3 viruses (H5N1), viruses were inactivated as previously described [27]. Cell supernatants were mixed with freshly prepared Triton X-100 to a final concentration of 1% (vol/vol) Triton X-100 and incubated for 1 h at room temperature. The inactivated supernatants were then transported out of the BSL3 to the BSL2 laboratory and used for NA assays as described above.

Potential interference of test molecules on the NA enzymatic activity was tested by incubating the A/Moscow/10/99 (H3N2) viral stock diluted in DMEM (10^7.8^ TCID_50_/mL final) with increasing concentrations of the test molecule (or DMEM for control) for 0.5 h at room temperature. Specific blanks were measured for each molecule. 25 µL were used for the NA test as described above and results were expressed as a ratio of corrected RFU of the sample to RFU of controls. Two independent experiments were performed in duplicate.

### 9 Viral growth assays in the presence of the molecules

For the viral growth assays in the presence of the molecules, A549 cells were seeded into 96-well plates at 0.15×10^5^ cells per well and cultured for 3 days to 100% confluence. Cells were then washed with DMEM and incubated with various concentrations of the different molecules diluted in infection medium (DMEM supplemented with 2 mM L-glutamine, 100 U/mL penicillin, 100 µg/mL streptomycin, 20 mM HEPES and 0.5 µg/mL trypsin). Six hours after treatment, cells were infected with influenza viruses at a moi of 2 or 0.2 by adding 25 µL per well of virus diluted in infection medium. Infection was allowed to proceed for 65 h at 37°C, 5% CO2 after which 25 µL of supernatant were collected for the NA activity test. Results are expressed as a ratio of corrected RFU of the sample to RFU of control (incubation with infection medium without test molecule). To check for cytotoxicity, viability assays were performed in parallel to each viral growth assay.

### 10 Test of infection efficiency after cell or virus pre-incubation with the molecules

A549 cells were seeded into 96-well plates at 0.15×10^5^ cells per well and cultured for 3 days to 100% confluence. For the 'Cell Preincubation' test, cells were washed with DMEM and incubated with various concentrations of the different molecules diluted in 200 µL per well of infection medium for 14 h. After two washings with DMEM, cells were infected with influenza A/Moscow/10/99 (H3N2) virus at a moi of 7 during 15 min and washed twice with infection medium. Infection was allowed to proceed for 5 h at 37°C. For the ‘Virus Preincubation’ assay, the molecules were diluted in infection medium and A/Moscow/10/99 (H3N2) viral stock (10^8.8^ TCID_50_/mL) was treated with increasing concentrations of the molecules for 14 h. Cells were then washed with DMEM and incubated for 15 min with the virus and molecule mix diluted 12 times. Infection was allowed to proceed for 5 h at 37°C. In both assays, the number of infected cells was estimated with a NA test. Cells were washed with PBS and lysed by shaking for 1 h with 25 µL per well of Triton 1X. The cell lysis extracts were used for a neuraminidase test as described above. Results were expressed as a ratio of corrected RFU of sample to RFU of control (solvent treated infections). Statistical significance was calculated in comparison to results for control cells using two tailed Welch t test.

### 11 EC_50_ and CC_50_ calculations

Viability and antiviral data were analyzed using the following three-parameter non linear logistic regression (l3) function [28]

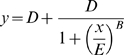



were y is the response, D is the upper limit (response when the dose x is ‘infinite’), E is denoted EC_50_ or CC_50_ and is the dose producing a response half-way between the upper limit and lower limit (0), and B is the relative slope around E. This model is the shortened form of the four parameter logistic function where the lower limit is fixed to 0. Results were obtained by fitting the l3 function using the package drc [29] in the R Statistical Language (version2.7.1). Parameters of the l3 model were estimated and fitted curves were plotted only if the data set contained one response <D/2.

## Results

### 1 Global transcriptional signature of influenza A infection

**Figure 1:**
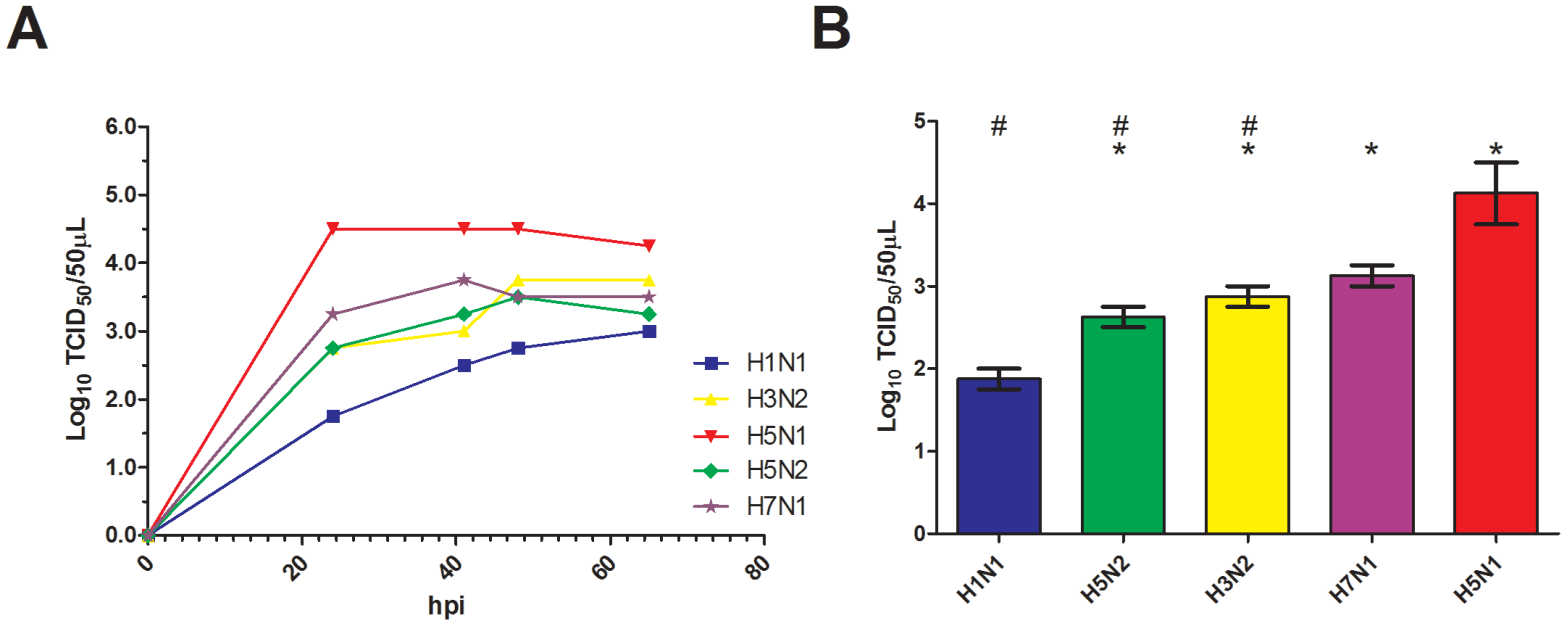
Comparison of viral replication kinetics between influenza viruses in A549 cells. A. A549 cells were infected at a moi of 0.1 with influenza virus A/New Caledonia/20/99 (H1N1), A/Moscow/10/99 (H3N2), A/Turkey/582/2006 (H5N1), A/Finch/England/2051/94 (H5N2), and A/Chicken/Italy/2076/99 (H7N1) and supernatants were collected at 24, 41, 48 and 65 hpi for end point titration assays. B. Each bar represents the mean of viral titers at 24 hpi for two independent experiments and titers were statistically analyzed by the one tailed Welch t-test; *: titers greater than H1N1 titers (p-value <0.05), #: titers lower than H5N1 titers (p-value <0.05).

To characterize the global cellular gene-expression response to influenza A infection, human pulmonary epithelium A549 cells were infected with human A/New Caledonia/20/99 (H1N1) and A/Moscow/10/99 (H3N2) and avian A/Turkey/582/2006 (H5N1), A/Finch/England/2051/94 (H5N2), and A/Chicken/Italy/2076/99 (H7N1) influenza viral strains. These viruses are herein referred to as H1N1, H3N2, H5N1, H5N2 and H7N1. A549 cells express both sialic acid α2,6- and α2,3-galactose receptors [30], [31] and were shown to be infected by human, avian and swine influenza viruses [32], [33]. Infections were performed at 37°C, a temperature at which both human and avian influenza viruses efficiently infect cell cultures [34] and at a moi of 0.1. In these conditions, there was evidence of productive viral replication of all viruses but with some kinetic and yield differences between viruses, as determined by infectious titers (TCID_50_) of supernatants of influenza virus infected A549 cells (Figure 1). The H5N1 virus titers peaked higher and earlier (24 hpi) compared to other viruses titers. Avian H7N1 and H5N2 viruses replicated with correct efficiencies, similar to the human H3N2 virus. In contrast, the human H1N1 virus strain replicated slower (titers peaked at 65 hpi) and grew to lower titers than other viruses (p-value <0.05 at 24 hpi).

To determine the host gene-response to infection, total cellular RNA was extracted at 24 hpi and submitted to reverse transcription in the presence of ^33^P. Each condition was performed in 5 independent replicates. All labeled cDNAs provided a good radioactive intensity and were hybridized onto home-made nylon microarrays containing 8782 IMAGE cDNA clones. All hybridizations were of good quality according to signals within acceptable range, number of features present, and signals from control spots.

**Figure 2:**
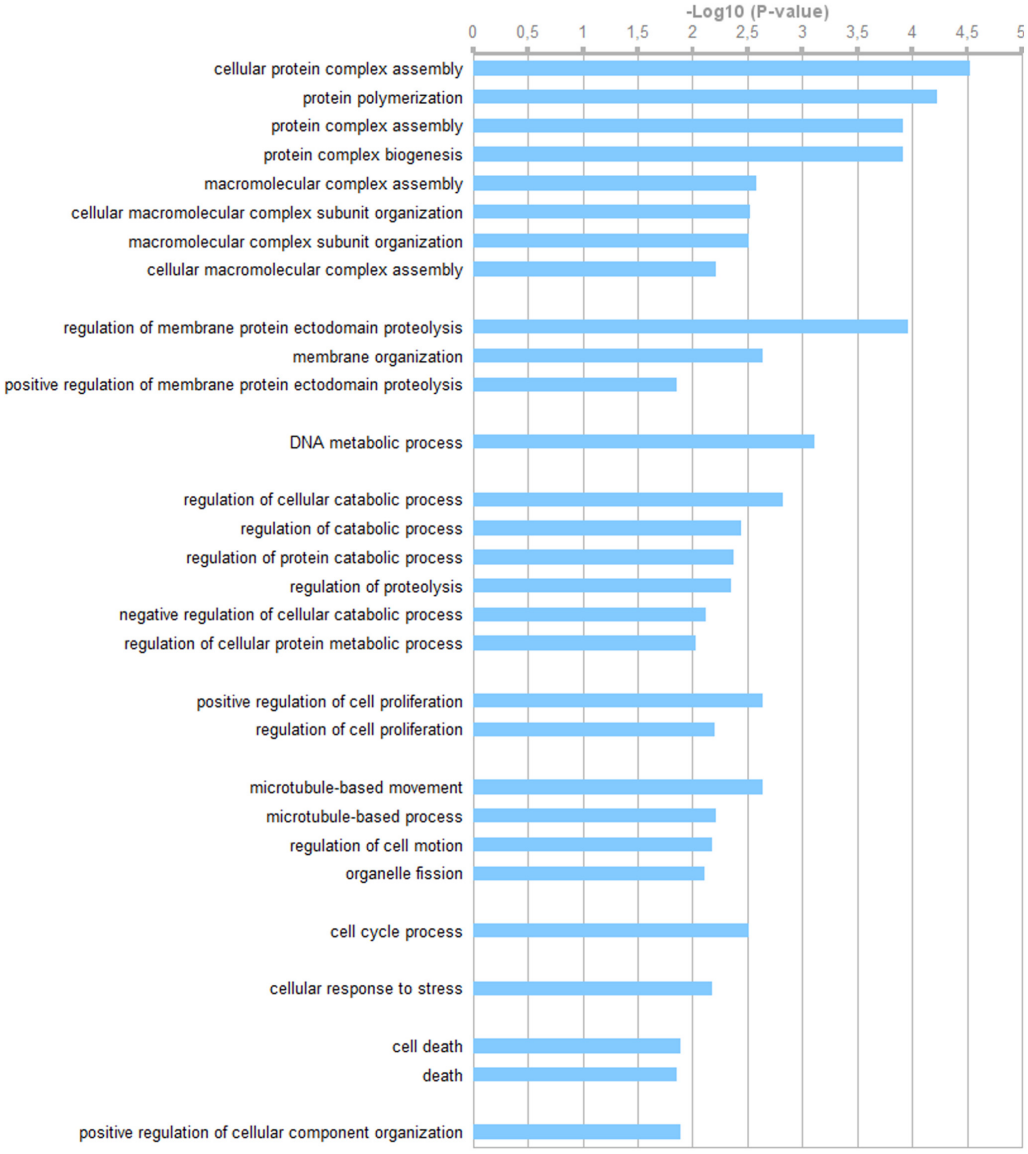
Gene enrichment analysis of the 300 genes of the infection signature. Discriminatory genes were analyzed by DAVID for associations with particular Gene Ontology terms. The negative Log_10_(P-values) of enriched terms (plotted in bar) refer to how significant an association a particular ontology term has with the gene list. The 30 most significant biological process are grouped according to their biological meaning.

Supervised analysis of normalized gene expression data was conducted using the SAM algorithm. This algorithm was used to identify genes whose expression levels were significantly altered by influenza infection. We set the delta threshold in the SAM analysis to allow an acceptable false discovery rate (FDR) of 10%. We found that the expression levels for a total of 300 genes (representing 3.4% of the genes considered present on the chip) differed significantly between mock and infected samples (Table S2). Using the DAVID Bioinformatics Resources database, we annotated this signature using the gene ontology (GO) terms. This revealed an enrichment of genes related to various cellular processes such as protein complex biogenesis, membrane and microtubule organization, DNA metabolic and catabolic processes, cell proliferation regulation, cell cycle and cell death (Figure 2).

**Figure 3:**
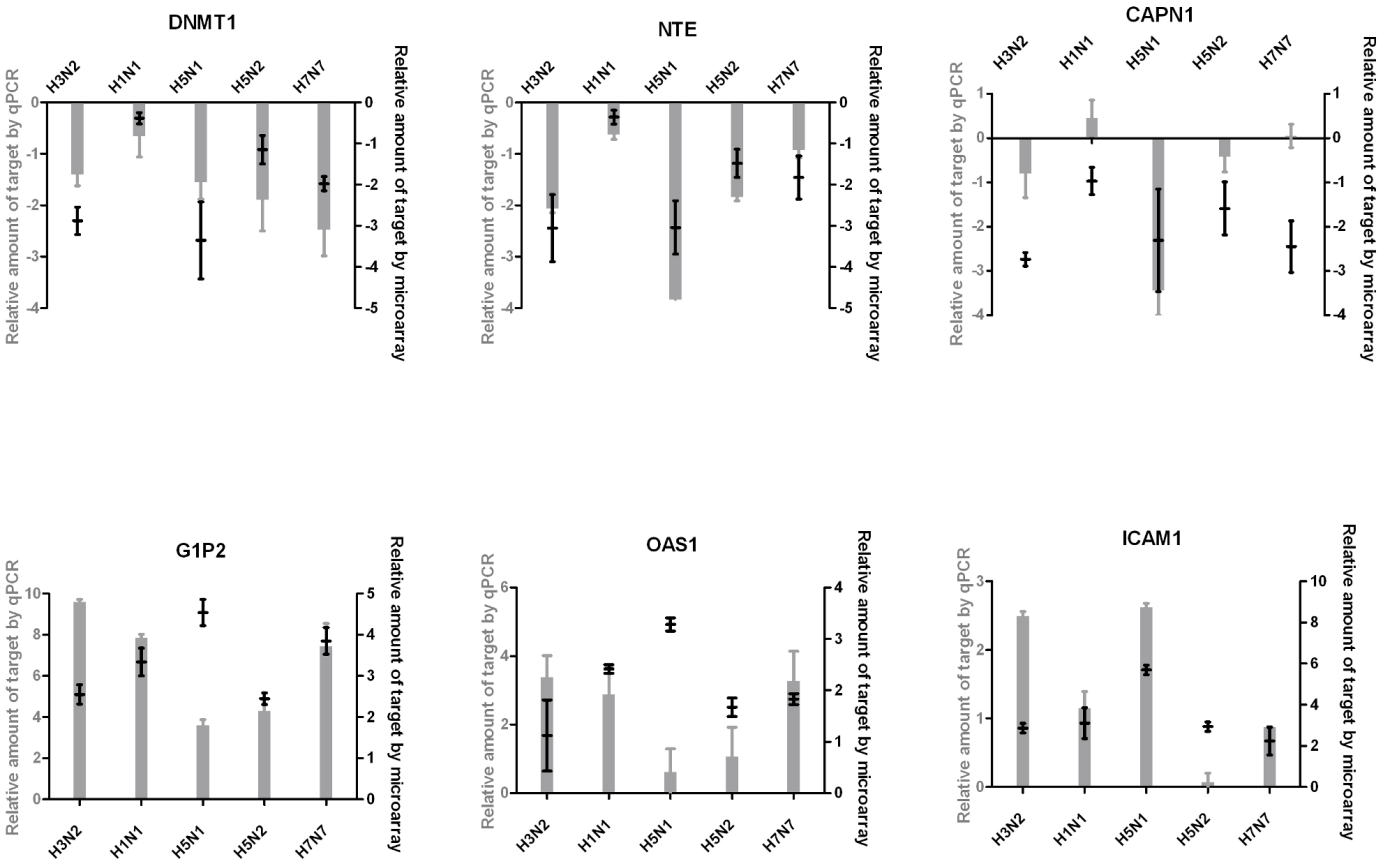
Validation of microarray results by real-time quantitative RT-PCR analysis. Expression of 6 genes from DNA microarray analysis (black cross) is compared with real-time quantitative PCR data (grey bar). Cross represent fold changes (mean ± S.D.) given by the microarray analysis  =  Log_2_ (normalized amount of target gene in infected samples) - Log_2_ (normalized amount of target gene in mock samples) (right). The formula used to determine the amount of target gene in infected cells by RT-qPCR, normalized to *GAPDH* and relative to mock is: 2^−ΔΔCt^ where Ct is the threshold cycle and ΔΔCt  =  (Ct target infected – Ct gapdh infected) – (Ct target mock – Ct gapdh mock). Bar represented Log2 expression level (mean±S.D.) of target genes in two independent quantitative RT-PCR analysis (left).

A subset of six genes with absolute fold changes in log_2_ (FCs) above 2 was selected to validate the microarray analysis by quantitative RT-PCR (RT-qPCR) analysis: *DNMT1*, *NTE* and *CAPN1* that were found downregulated in infected cells and *G1P2*, *OAS1* and *ICAM1* that were upregulated. The 6 genes were chosen at random among the most 20 dysregulated genes upon infection. This quantification was performed on new samples equivalent to those used for the microarray analysis. Figure 3 shows the confirmation by RT-qPCR of the microarray data. For each gene and each strain, microarray FCs are presented as a black boxplot and RT-qPCR results are depicted as a gray histogram. Results from RT-qPCR were in good agreement with the cDNA microarray analyses for five out of six genes tested. Indeed, except for *CAPN1* (p-value = 0.1), significant difference between infected and non infected cells was also observed in quantitative RT-PCR analysis (p-value <0.05, Welch t-test), similar to DNA microarray analysis. This result was acceptable considering that samples analyzed by RT-qPCR were different from those used in the microarray analysis.

**Figure 4:**
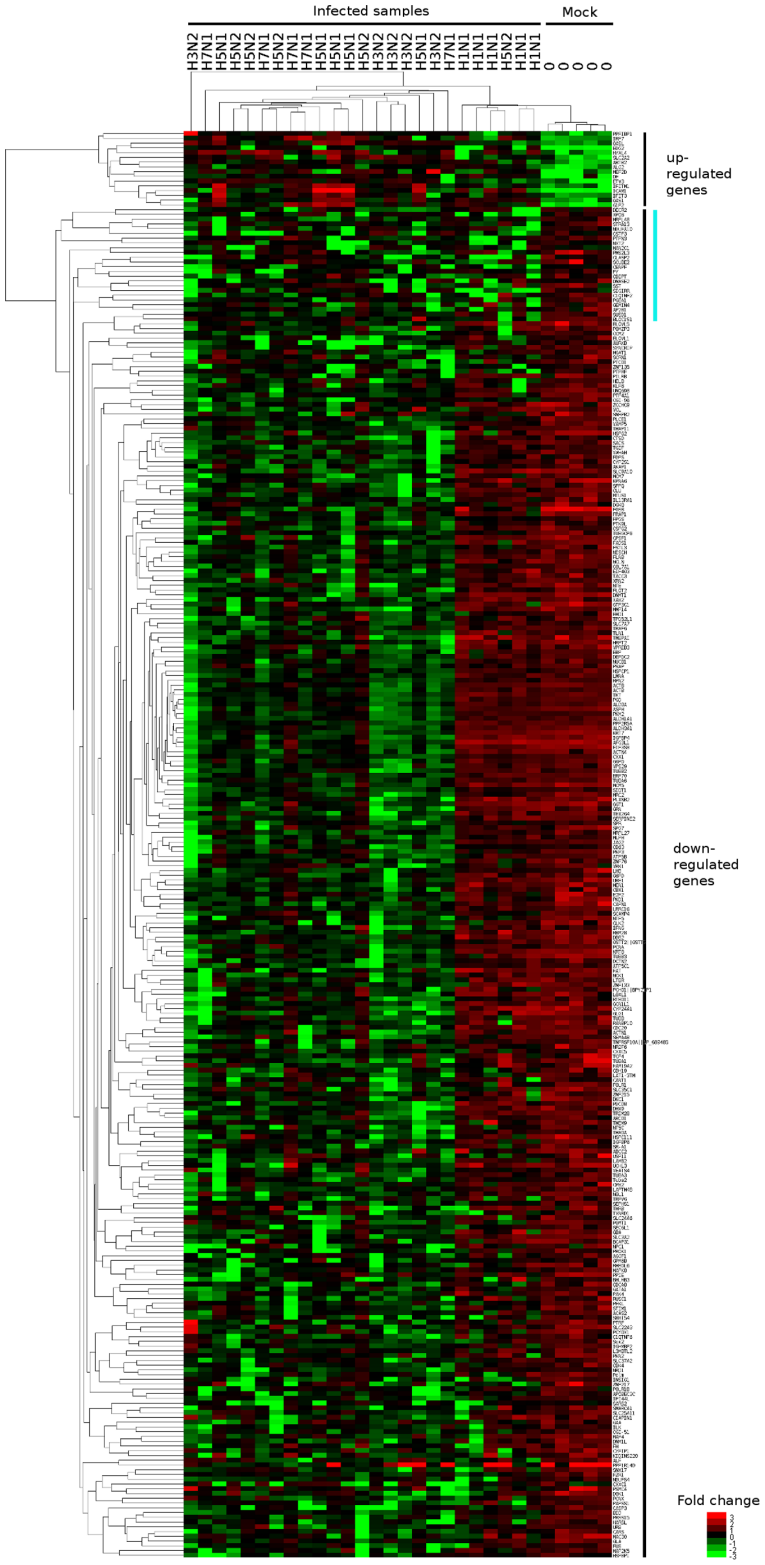
Hierarchical clustering and heatmap of the 300 genes that discriminate mock and infected samples. Heatmap representing the expression levels of the 300 genes differentially expressed between infected and mock cells. Red squares indicate the expression levels above the median of the gene abundance across the sample and green squares the expression levels below. The median values were clustered hierarchically in both dimensions. Dendograms indicate the correlation between groups of samples (horizontal) and genes (vertical). Vertical lines in the right portion indicate the 2 distinct gene expression patterns: up and down-regulated genes during infection. The blue line stands for the genes down-regulated in samples infected with H1N1 influenza virus. Gene expression data for the 300 genes are reproduced in table S2.

To visually compare the changes in mRNA abundance for the 300 genes found to be influenced by influenza infection, hierarchical clustering analysis in both dimensions was performed. Results are depicted in the heatmap representation of Figure 4. Dendrograms indicate the correlation between samples and genes. We verified that mock samples were sorted together vs infected ones. The H1N1 samples co-clustered with the mock samples suggesting that infection with this strain induced few gene expression changes. We verified this result by conducting a virus-specific SAM analysis on the mock vs one virus samples. For a FDR of 10%, only 36 genes were found to be regulated by H1N1 infection in comparison to 2298 genes by H3N2, 1510 by H5N2, 3020 by H7N1 and 1455 by H5N1. The main difference between H1N1 and other viruses lay in the number of down-regulated genes during infection. Whereas H3N2, H5N1, H5N2 and H7N1 influenza viruses induced a down-regulation of most of the genes tested, a similar number of genes were down- and up-regulated by H1N1 (highlighted by the blue vertical line in the heatmap Figure 4). As H1N1 viral titer was lower at 24 hpi than titers of other viruses (Figure 1B), the scope of gene-expression changes induced upon infection correlated, at least partially, to the viral replication efficiency of the virus-cell system used in this study.

Interestingly, out of the 300 genes of the global infection signature, only 16 were upregulated in all infected cells. These 16 genes were associated to three GO biological process, including two related terms, “viral reproductive process” and “viral reproduction”, that annotate genes encoding proteins involved in the virus life cycle. Two genes were associated to these terms: *ICAM1*, which is the major receptor for human rhinovirus [35], and *IRF7*, which activates the expression of Epstein-Barr Virus Latent Membrane Protein 1 [36] (p-value = 0.07 and 0.08, respectively). While *IRF7* has not been directly involved in influenza virus life cycle yet, *ICAM1* was recently identified as a proviral factor that may be co-opted by influenza virus [37]. The third associated biological process was the term “immune response” (p-value = 0.04) annotating 4 genes (*ICAM1*, *OASL*, *OAS1* and *CFD).* Therefore, the upregulated genes were mostly associated with the immunological response. Besides, seven of the 16 genes were interferon stimulated genes (ISGs): *IFITM1*, *ICAM1*, *IFIT3*, *OAS1*, *G1P2* (or *ISG15*), *IRF7* and *OASL*. These results were in accordance with previous studies showing the upregulation of immune response associated genes in samples infected in vitro and in vivo with various influenza viruses [9], [11], [12], [37], [38], [39], [40]. Gene expression levels in each group of samples are depicted in Figure S1. All ISGs were markedly more up-regulated in H5N1 infected cells than in other samples. This hyperstimulation has been described in other transcriptional studies [11], [40], [41] reinforcing the validity of the experimental cell-virus system developed in the present study.

### 2 *In silico* drug screening of the Connectivity Map

The Connectivity Map is a collection of genome-wide transcriptional expression data from cultured human cells treated with bioactive small molecules [13]. The associated website provides tools to find molecules connected to the query signature i.e. any list of genes associated with a biological test. The similarity of the query signature to each of the reference expression profiles is assessed and quantified by a normalized score, from -1 for a molecule that reverses the signature to +1 for a molecule which induces gene expression changes similar to the query signature.

Our strategy was to query the Connectivity Map with a list of genes differentially expressed in infected cells to find molecules that induced the opposite gene expression changes. We hypothesized that such molecules may influence host cell metabolism in such a way that effective viral replication would be altered.

**Figure 5:**
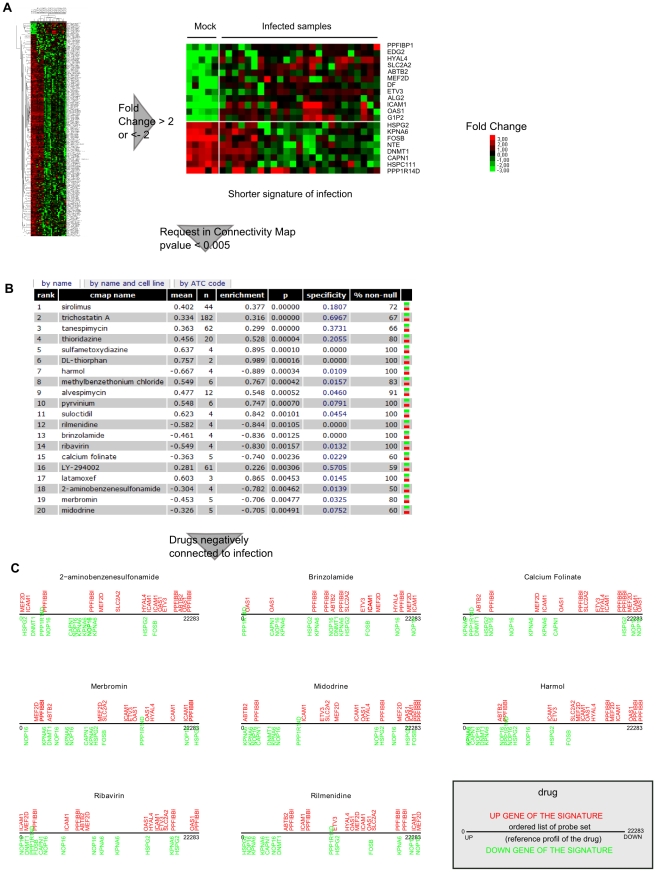
Gene expression-based screening identifies eight potential antiviral molecules. A. List of cellular genes chosen to query the Connectivity Map. A circumscribed signature of infection was derived from the 300 genes discriminating mock and infected samples by selecting genes with a fold change > 2 or <−2. The fold change is defined as the ratio of the mean gene expression in infected samples to the mean for the corresponding mock infection in log_2_. This selection resulted in a list of 20 genes, 12 being up-regulated during infection and 8 down-regulated (see Table 1). These genes constituted the signature used to query the online database Connectivity Map. B. Drugs with significant enrichment to influenza virus infections in the Connectivity Map. Significance cut-off was set at p-value <0.005. The permutation p-value estimates the likelihood that the results would be observed by random chance. Mean: the arithmetic mean of the connectivity scores for the post-dose changes (or instances) by the given molecule; n: the number of instances of a given molecule in the CMAP database; Enrichment: a measure of the enrichment of those instances in the order list of all instances, Positive enrichment scores are of interest if perturbagens inducing the biological state represented by the signature used to produce the result are sought. Likewise, if reversal or repression of the biological state encoded in the query signature is required, perturbagens with negative enrichment scores are of interest. The specificity value is defined as the frequency at which the enrichment of a set of instances equals or exceeds that of the same set of instances in queries executed on 312 published, experimentally derived signatures using the Molecular Signatures Database. Lower values are associated with a greater specificity; the non null percentage represents a measure of the support for the connection between a set of instances and a signature of interest based on the behavior of the individual instances in that set. C. 8 molecules are negatively connected to influenza virus infection (p-value <0.005). A graphical representation of the location of the signature of infection is depicted for each molecule, taking the instance with the most negative connectivity score of each molecule. The x-axis represents the genes of the expression profile of the molecule, rank ordered according to their differential expression relative to the control. The location of each gene of the infection signature is appreciated along the x-axis.

A critical step in this screening was to define the query signature. As the number of upregulated genes was very low (5.3%) in the list of 300 genes defined by the analysis, a lack of specificity resulting from a loss of information for up-regulated genes could be introduced in drug selection if the signature was not corrected for this bias. By selecting genes with the most drastic changes in level of expression (fold change in log_2_ > 2 or <−2), we were able to define a signature of 20 genes for influenza A virus infection with similar amounts of those up and down regulated (Figure 5A, Table 1).

**Table 1:** Genes of the circumscribed signature of infection.

Gene	Gene symbol	I.M.A.G.E. CloneID	Fold Change
***Transcription Regulation***			
Ets variant gene 3	**ETV3**	1473929	7.24
Myocyte enhancer factor 2D	**MEF2D**	4209	5.13
FBJ murine osteosarcoma viral oncogene homolog B	**FOSB**	79022	−2.69
DNA (cytosine-5-)-methyltransferase 1	**DNMT1**	768241	−2.06
Ankyrin repeat and BTB (POZ) domain containing 2	**ABTB2**	204456	2.28
***Immune response***			
ISG15 ubiquitin-like modifier, G1P2	**ISG15**	742132	3.48
2′,5′-oligoadenylate synthetase 1, 40/46kDa	**OAS1**	666703	2.07
Complement factor D (adipsin), DF	**CFD**	666128	2.99
***Cell surface protein***			
Patatin-like phospholipase domain containing 6, NTE	**PNPLA6**	431990	−2.01
Heparan sulfate proteoglycan 2	**HSPG2**	3339	−2.23
Solute carrier family 2 (facilitated glucose transporter)	**SLC2A2**	207963	2.23
PTPRF interacting protein, binding protein 1 (liprin beta 1)	**PPFIBP1**	263094	2.04
Intercellular adhesion molecule 1 (CD54)	**ICAM1**	3383	3.36
Lysophosphatidic acid receptor 1	**LPAR1**	505524	3.63
***Enzyme***			
Hyaluronoglucosaminidase 4	**HYAL4**	668088	2.90
Asparagine-linked glycosylation 2 homolog	**ALG2**	150443	2.35
Calpain 1, (mu/I) large subunit	**CAPN1**	70555	−2.20
Protein phosphatase 1, PP1	**PPP1R14D**	140525	−3.13
***Nuclear protein***			
Karyopherin (importin) alpha 6	**KPNA6**	16650	−2.21
Hypothetical protein HSPC111	**NOP16**	505242	−2.08

Fold Change  =  log_2_(Inf/Mock).

By querying the connectivity map with this concise signature, we obtained c-scores for 6100 instances, representing more than 1000 molecules in various conditions [14]. We selected those associated with the most strongly anticorrelated signatures (negative enrichment) and which had a p-value less than 0.5% (Figure 5B and 5C). Applying this filtering step left us with eight candidate molecules: harmol, rilmenidine, brinzolamide, ribavirin, calcium folinate, 2-aminobenzenesulfonamide, merbromin and midodrine (from the most negatively correlated to the least negatively correlated drug). The relevance of our selection was supported by the fact that ribavirin, an already known influenza virus inhibitor, was identified with a negative enrichment of -0.83 and a pvalue of 0.00157. Except for the topical antiseptic merbromin, the other selected molecules have various therapeutic indications (depicted in Table S3) but are not referenced as antivirals.

Graphs in Figure 5C report how the different genes of the infection signature behave in the expression profile of the selected molecules. Although the genes down-regulated during infection are generally up-regulated in response to the molecule and conversely the up-regulated genes of the signature are globally down-regulated by the molecule, none of the molecules available in this data bank were able to completely reverse the infection signature.

### 3 Evaluation of the antiviral potency of the selected drugs on H3N2 viral growth

We assessed the effect of the eight selected molecules on influenza replication in vitro. Cell viability, as assessed by the neutral red assay, and viral growth, as quantified by a neuraminidase (NA) activity test, were conducted in parallel. Before using the NA activity test as an indirect measurement for viral impairment, we checked firstly that the different influenza viruses used in this study had sufficient neuraminidase activities to be quantified using this method (Figure S2A). For all tested viruses and for a signal to background ratio between 2 and 70, the fluorescence was proportional to the amount of virus present (TCID_50_/mL). During the evaluation of the drug panel, all signal to background ratios were included between 2 and 70. Secondly, we controlled that the different molecules did not inhibit the enzymatic activity of NA to be sure that a drop in RFU would only reflect a drop of viral titer. While concentrations of merbromin above 50 µM and harmol above 500 µM inhibited NA activity, incubation of the virus with increasing concentrations of the molecules otherwise resulted in no inhibition (Figure S2B). Therefore, for these two molecules below these concentrations and for other molecules of the drug panel, viral growth can be assessed by a neuraminidase test.

Evaluation of the drug panel was first conducted on influenza A/Moscow/10/99 (H3N2) virus. A549 cells were incubated with increasing concentrations of the molecule for 6 h before infection. This time was chosen based on the duration of treatment indicated in the Connectivity Map to obtain similar cellular response before infection [14]. Infection was allowed to proceed for 65 h which represents multiple cycles of infection, however similar results were observed at 24 and 48 hpi (Figure S3).

The viability data of five independent experiments are given in Figure S4. The 50% cytotoxic concentrations (CC_50_) were determined by regression analysis. The CC_50_ of calcium folinate, 2-aminobenzenesulfonamide and midodrine could not be determined since none of these molecules was cytotoxic at the highest tested dose.

The effect of each of the molecules on viral growth was tested using the H3N2 virus at a moi of 0.2 and 2. Dose-response curves were fitted by regression analysis (Figure S5) and used to determine the 50% effective concentration (EC_50_) of each molecule if at least one response was inferior to 50%. Selective indexes (SI) were calculated as CC_50_/EC_50_ and used to classify selected molecules as inactive (SI<2), weak inhibitors (2<SI<10), moderate inhibitors (10<SI<50) and strong inhibitors (SI>50) (Figure 5). In agreement with previous observations [42], we noted that SI were dependent on the moi, since molecules are more effective at lower moi. In our conditions, at a moi of 0.2, two molecules (calcium folinate and 2-aminobenzenesulfonamide) were ineffective, two (harmol and merbromin) were weak inhibitors, two (brinzolamide and midodrine) were moderate inhibitors and one (ribavirin) was a strong inhibitor. At a moi of 2, whereas brinzolamide was reclassified as a weak inhibitor, the other molecules remained in the same class despite their SI being weaker. As an example, the CC_50_ for midodrine was superior to 4250 µM and EC_50_ was comprised between 322 µM (moi of 0.2) and 532 µM (moi of 2). Concerning rilmenidine, which was dissolved in DMSO, it was not possible to conclude on an effect. DMSO has previously been shown to be cytotoxic and to inhibit influenza infection above 4% (vol/vol) [43] however it is still used as a major solvent for molecules in high-throughput screening. In this study, the CC_50_ for DMSO was 2.9% (v/v) - the concentration used to obtain 1550.7 µM of rilmenidine- and the EC_50_ was comprised between 1.0% (moi of 0.2) and 1.8% (moi of 2). The EC_50_ of rilmenidine was significantly different from that of DMSO at a moi of 2 (p = 2e−4) but not at a moi of 0.2 (p = 0.85).

However, even if this molecule is considered ineffective against the H3N2 influenza virus, we did obtain a very high confirmation rate (62.5%) in comparison with the hit rate of classical high-throughput screening (3.2% in [43]). This clearly indicates that our in silico screening was effective and strongly supports its power at selecting the antivirals: harmol, merbromin, brinzolamide, midodrine and ribavirin.

### 4 Antiviral effects of most of the molecules are due to an action on cells rather than on viruses

Molecules selected by the in silico screening were chosen from the Connectivity Map based on the gene expression changes they induce in treated cells. To check that the antiviral properties of the five efficient molecules were actually mediated by an action on cells and not by an indirect effect on the virus, we conducted two assays in parallel in which either the cells or the H3N2 virus were preincubated with a series of concentration of the molecules. The efficiencies of infection were estimated by measuring the neuraminidase activity associated to cells at an early time of infection. In the preincubated cells assay, cells were in contact with molecules for 14 hours before being infected with H3N2 virus without any drugs. As the cells were washed twice before infection, we assumed that the virus should not be in contact with the molecules during infection. Thus the molecules should not alter the viral structure nor change parameters playing a direct role on viral entry (as the extracellular pH for example). Consequently an inhibition of infection in this assay would mean that the molecule had an effect on cells. In contrast, in the preincubated virus test, the viral stock was treated with the molecules during 14 hours while the cells were in contact with molecules though after dilution and for only 15 minutes during infection. We assumed that this exposure time and molecule concentrations were too low to induce any effect on the cells. If a molecule should inhibit viral growth by altering the functional properties of the virus (viral structure or surface glycoprotein), infection would be inhibited in the preincubated virus condition but not in the preincubated cells one.

**Figure 6:**
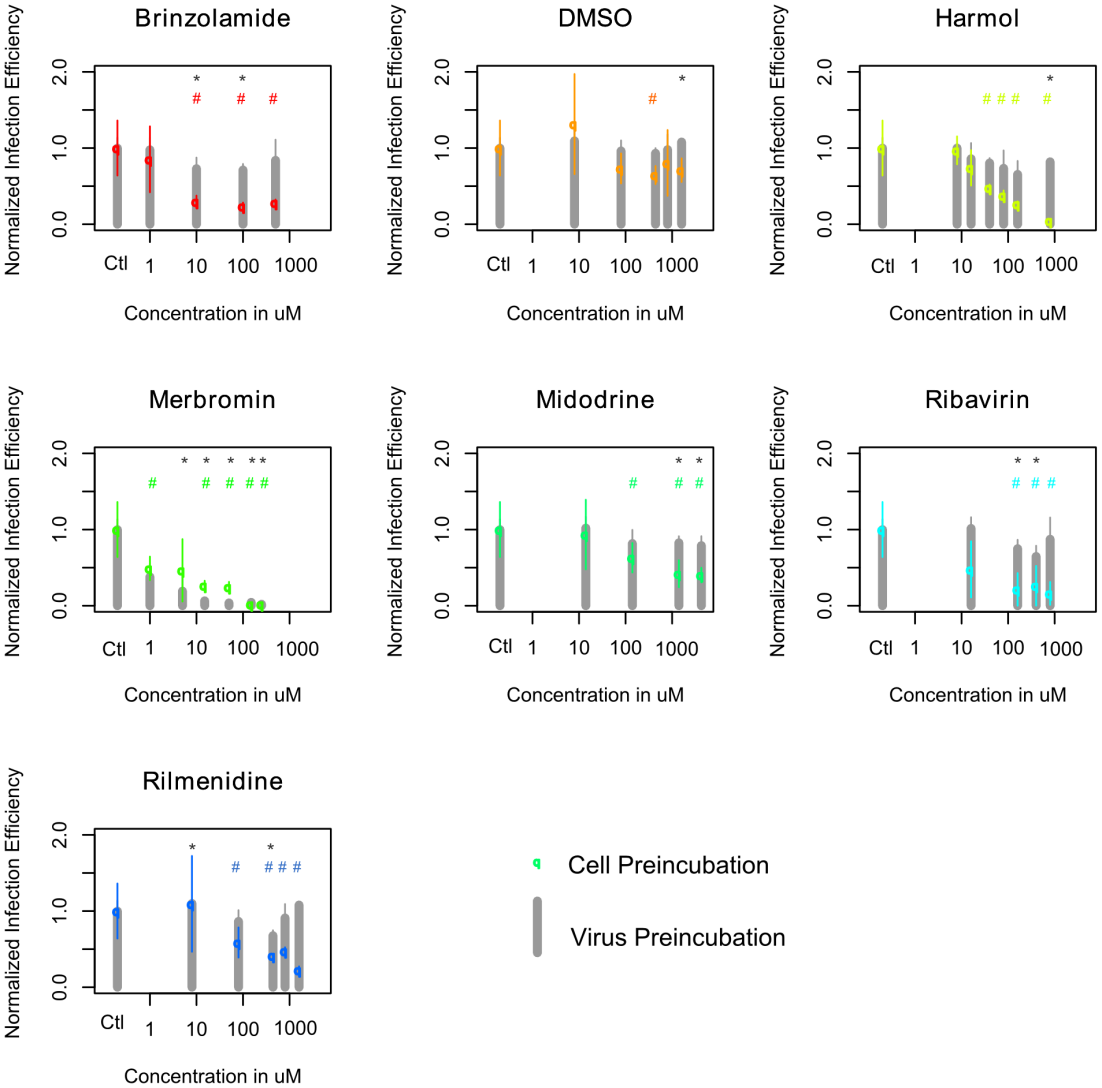
Antiviral effects of most of the molecules are due to an action on cells rather than on the virus. In the ‘Cell Preincubation’ assay, A549 cells were treated with increasing concentrations of the molecules or solvent for 14 h and washed twice before incubation with H3N2 influenza A virus at an moi of 7 during 15 min and infection for 5 h in medium without drug. In the ‘Virus Preincubation’ assay, H3N2 viral stock was treated with increasing concentrations of molecules or solvent for 14 h and diluted 12 times before incubation with cells during 5 min and infection for 5 h. In both assays, the number of infected cells was estimated with a neuraminidase test after cell lysis with Triton 1X. The normalized infection efficiency was calculated as RFU in treated cells/RFU in control cells. Values represent the mean of two independent experiments (+/− standard deviation). Results of the preincubated cell test are plotted with colored dots and results of the virus preincubated assay are depicted with grey bars. * and # indicate statistically significant differences of infection efficiency in comparison to untreated control cells (Welch two-sample t test, pvalue <0.05), for the preincubated cell test and for the preincubated virus test, respectively.

Results of both tests for the five efficient molecules are depicted in Figure 6. After preincubating the viral stock with the molecules, a few infection efficiencies were significantly different of the control (p-value <0.05, two tailed Welch t-test). However, except for merbromin, infection efficiencies after virus preincubation were included between 64% (for ribavirin c = 400 µM) and 110% of the control (for rilmenidine c = 8 µM). Therefore, the different drugs exerted very limited effects on the virus. In contrast, statistically significant inhibitions of infection efficiency were noted after cells preincubation with each molecule at higher concentrations (above 10 µM for brinzolamide, 40 µM for harmol, 1 µM for merbromin, 140 µM for midodrine, 160 µM for ribavirin and 80 µM for rilmenidine). Infection efficiency decreased to 23% for brinzolamide (100 µM), 5% for harmol (800 µM), 2% for merbromin (250 µM), 40% for midodrine (1400 and 4250 µM), 26% for ribavirin (800 µM) and 23%3 for rilmenidine (1600 µM). We concluded from these tests that the antiviral effect of these molecules is due to an action on cells rather than on the virus. Merbromin on the other hand inhibited viral infection in both assays. This was not surprising since this molecule is a topical antiseptic known to inactivate influenza viruses [44]. However, our results indicate that this molecule may also inhibit viral replication through a cellular effect.

### 5 None of the molecules which are positively correlated to the infection signature, impaired H3N2 influenza viral growth

In order to control that the antiviral effect of the molecules is specifically associated with inversion of the infection signature, we assessed the effect of some molecules positively correlated to the signature. Seven drugs, alvespimycin, DL-Thiorphan, latamoxef, methylbenzethonium chloride, pyrvinium, sulfameter (or sulfametoxydiazine) and sulodictil, were chosen according to the following criterion: p-value <0.5%, mean > 0.35 and a specificity <0.1 (Figure 5B).

Viability and viral growth assays were performed on A549 cells infected with H3N2 virus at a moi of 0.2 and 2, as described for negatively correlated drugs. Dose-response curves (Figure S6 and S7) were used to determine CC_50_ and inhibitory EC_50_ (Table S4). In these conditions, inhibitory SI were lower than 2, or than SI of DMSO for DL-Thiorphan and Sulodictil. Thus none of the positively correlated drugs inhibited viral replication at both moi. In contrast, four drugs (alvespimycin, methylbenzethoniumchloride, pyrvinium and sulodictil) enhanced viral production at a moi of 0.2. Increase of viral titers (TCID_50_/mL) was up to 2 log_10_ and was statistically significant for alvespimycin, methylbenzethoniumchloride, and sulodictil 40 µM (p-value <0.05, two tailed Welch t-test). Therefore, these results strengthen our hypothesis that modulation of host cell transcription may have an impact on viral replication.

### 6 Some antivirals are effective against a broad range of influenza A virus strains, including the pandemic H1N1 influenza virus

**Figure 7:**
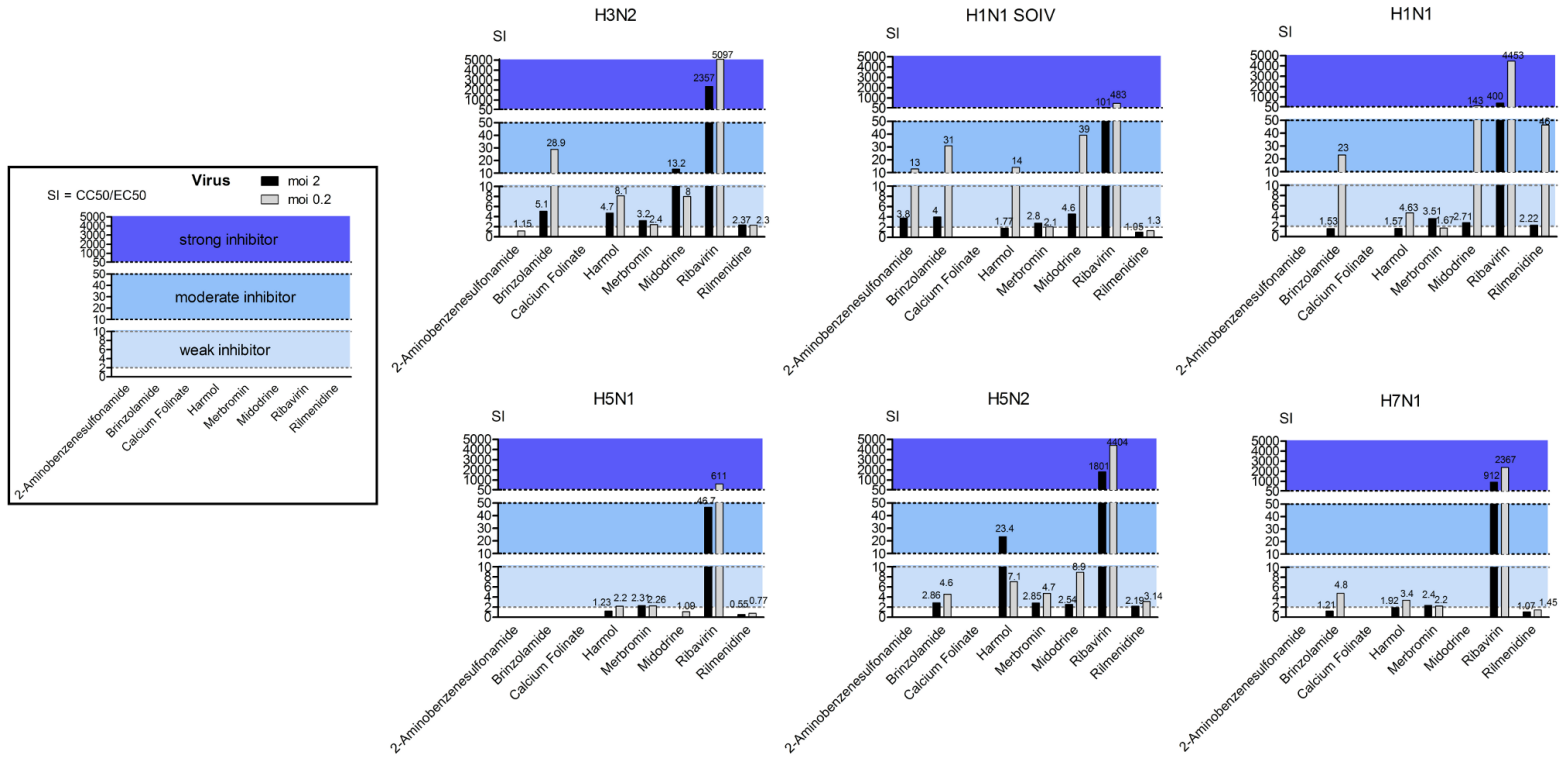
Antiviral potency of the 8 negatively correlated molecules on different influenza A viruses (moi 0.2 and moi 2). Selective indexes (SI) were calculated as CC_50_/EC_50_ (Tables 2 and 3) and used to classify molecules as inactive (SI<2), weak inhibitors (2<SI<10), moderate inhibitors (10<SI<50) and strong inhibitors (SI>50).

We hypothesized that one advantage of our gene-expression based screening is that the selected molecules would have an activity against various influenza A viruses. Indeed, since we selected a gene signature of infection common to different human and avian strains, we assumed this as a prevailing cellular response to many influenza viruses. Therefore, we tested the effect of the selected molecules on the viral growth of the different strains used for the initial microarray analysis, i.e A/New Caledonia/20/99 (H1N1), A/Turkey/582/2006 (H5N1), A/Finch/England/2051/94 (H5N2), and A/Chicken/Italy/2076/99 (H7N1). Two independent assays in duplicate (4 replicates in total) for each virus were conducted in the conditions previously defined for the H3N2 virus. EC_50_ and SI were determined for each molecule and are summarized in Table 2, Table 3 and Figure 7. Molecules that inefficiently inhibited growth of the H3N2 strain (2-aminobenzenesulfonamide and calcium folinate) were also inefficient against other tested viruses. Conversely, the strongest H3N2 inhibitor, ribavirin, was also classified as a strong inhibitor of all viruses tested. However, ribavirin obtained different SI depending on the viral strain tested, allowing the viruses to be classified according to their sensitivities to this molecule: H3N2 > H5N2 and H1N1 > H7N1 > H5N1. Other drug screening tests carried out previously on MDCK cells (with an moi of 0.001) had already reported a higher sensitivity of H3N2 viral strains compared to H1N1 [42], [45]. In our tests, ribavirin EC_50_ was comprised between 6 µM (for H1N1 and H3N2 with an moi of 0.2) and 632 µM (for H5N1 with an moi of 2) in concordance with previously published results [42], [45]. Midodrine, the second most active molecule against the H3N2 strain, also showed an antiviral effect against both H1N1 (SI >2.7 for moi 2 and SI >142.5 for moi 0.2) and H5N2 (SI >2.5 for moi 2 and SI >8.9 for moi 0.2) viral strains. Brinzolamide was a moderate inhibitor of human H3N2 and H1N1 influenza viruses and a weak inhibitor of avian H5N2 and H7N1 influenza viruses. Harmol weakly inhibited all viruses tested, as did merbromin the EC_50_ for which were near to 50 µM, a concentration noted to interfere with the neuraminidase activity test. Finally, rilmenidine had an obvious antiviral effect on the H1N1 strain. Some of the molecules identified by our approach were therefore able to inhibit viral growth of all the viruses used to define the gene expression signature of infection.

**Table 2:** Potency of the inhibitors against human influenza A viruses in A549 cells.

		H1N1 moi 2	H1N1 moi 0.2	SOIV moi 2	SOIV moi 0.2	H3N2 moi 2	H3N2 moi 0.2
	CC50 (μM)	EC50 (μM)	EC50 (μM)	EC50 (μM)	EC50 (μM)	EC50 (μM)	EC50 (μM)
**2-Aminobenzenesulfonamide**	>6900	>690	>690	1821.67	533.83	>6900	6020
**Brinzolamide**	665.51	435.06	28.95	166.65	21.55	131.14	23.00
**Calcium Folinate**	>2400	>800	>800	>2400	>2400	>2400	>2400
**Harmol**	94.98	60.36	20.53	53.74	6.76	20.07	11.68
**Merbromin**	103.80	29.55	61.98	37.53	>50	32.38	42.87
**Midodrine**	>4250	1566.7	29.82	929.96	108.79	321.99	531.53
**Ribavirin**	29528	73.79	6.63	291.52	61.08	12.53	5.79
**Rilmenidine**	1125.3	506.22	24.22	1073	864.48	475.55	489.87
**DMSO**	1550.7	>1600	173.63	606.40	344.03	957.2	554.39
**p-value (Rilmenidine, DMSO)**	p = 0.0026	p = ND	p = 7e−04	p = 0.6128	p = 0.3552	p = 2e−4	p = 0.88

CC_50_: molecule concentration of 50% cytotoxicity; EC_50_: molecule concentration of 50% inhibition of viral replication; p-value: t-statistic for testing Rilmenidine values equal to DMSO values.

**Table 3:** Potency of the inhibitors against avian influenza A viruses in A549 cells.

		H5N1 moi 2	H5N1 moi 0.2	H5N2 moi 2	H5N2 moi 0.2	H7N1 moi 2	H7N1 moi 0.2
	CC50 (μM)	EC50 (μM)	EC50 (μM)	EC50 (μM)	EC50 (μM)	EC50 (μM)	EC50 (μM)
**2-Aminobenzenesulfonamide**	>6900	>690	>690	>690	>690	>690	>690
**Brinzolamide**	665.51	>500	>500	232.40	145.61	551.05	139.19
**Calcium Folinate**	>2400	>800	>800	>800	>800	>800	>800
**Harmol**	94.98	77.49	43.36	4.06	13.45	49.35	27.96
**Merbromin**	103.80	44.99	45.98	36.45	21.93	43.70	46.74
**Midodrine**	>4250	>4250	3896.3	1670.7	475.58	>4250	>4250
**Ribavirin**	29528	632.27	48.36	16.40	6.71	32.39	12.47
**Rilmenidine**	1125.3	2037.7	1466.8	514.32	358.69	1050.4	777.85
**DMSO**	1550.7	>1600	1829.6	733.6	564.1	1109.3	801.08
**p-value (Rilmenidine, DMSO)**	p = 0.0026	p = ND	p = 0.40	p = 2e−04	p = 0.1881	p = 0.53	p = 0.9

CC_50_: molecule concentration of 50% cytotoxicity; EC_50_: molecule concentration of 50% inhibition of viral replication; p-value: t-statistic for testing Rilmenidine values equal to DMSO values.

To determine if this strategy identified broadly effective influenza antivirals which could be active against emerging influenza viruses, we tested their effect on the viral growth of the recent pandemic H1N1 virus (referred to as H1N1 SOIV) (Figure S8). Interestingly, in comparison with A/New Caledonia/20/99 (H1N1) virus, a weak to moderate antiviral effect was observed for 2-aminobenzenesulfonamide whereas rilmenidine was ineffective. The other molecules had comparable effects on the two H1N1 virus strains, with brinzolamide, midodrine and ribavirin being the most effective antivirals. The EC_50_ of ribavirin were comprised between 61 µM (for an moi of 0.2) and 292 µM (moi of 2) revealing a resistance to this molecule that was 4 (moi 2) to 10 (moi 0.2) times more in the H1N1 SOIV strain compared to the H1N1 strain (pvalue = 3.6e−6 and 0.0012 respectively).

We compared drug sensitivities to viral growth curves of different viruses after infection of A549 cells at two moi (Figure S9). Viruses with good replication efficiencies and the faster kinetics (H5N1 and H7N1) were the most resistant to the drug panel. In contrast, selected antivirals had a better effect on delayed replication viruses (H1N1 and H1N1 SOIV). Drug sensitivities therefore partially correlated with viral growth kinetics. However, some strain specificity may also account for drug sensitivities. Indeed, H3N2 virus was one of the most drug sensitive virus, while replicating as efficiently than H7N1 virus (but a bit slower).

To conclude, five molecules out of the eight potential molecules selected by our in silico screening inhibited viral growth of the H1N1 SOIV, a virus that was unknown when we first defined the signature of infection and queried the Connectivity Map. These results are promising and strongly indicate that this approach identifies molecules with a broad anti-influenza spectrum of activity.

## Discussion

### The virally induced gene-expression signature

Influenza infection induces various intracellular signaling cascades and important downstream gene expression host-cell modifications [46]. Despite their host-range restriction that may reflect the better adaptation to host factors [47], all influenza A viruses can infect the same cells in vitro, prompting us to assume that they may hijack common cellular proteins for their own replication. This is the first study to compare the cellular gene expression modifications induced by five different influenza A virus subtypes. As already described in previous transcriptional in vitro [40] and in vivo studies [11], [12], we found that H5N1 infection induced a strong upregulation of interferon response genes. This sustained hyperinduction has been correlated with the high virulence of this virus in animal models [11], [12]. In patients, H5N1 infection results in a massive production of cytokines and chemokines, referred to as the cytokine storm, which could be responsible for the severity of the disease [48]. Here we observed that H5N1 induced the expression of more, and to a greater extent, inflammatory/immune response genes than any of the other subtypes. Molecular mechanisms supporting the higher activation of interferon signaling by H5N1 in comparison with other subtypes remain undetermined. In contrast, we found that A/New Caledonia/20/99 (H1N1) infection leads to the smallest change in gene expression at 24 hpi. One could speculate that H1N1 virus, as a human influenza virus, would be well adapted to human A549 cells and could replicate in these cells with basal level of proteins, thus without having to induce much gene-expression changes. However a well adapted virus would efficiently replicate in these cells. We performed replication kinetics in A549 cells with the different viruses and observed that H1N1 virus grew to lower titers than other viruses. Two hypothesis can be formulated to explain the correlation between the weak growth of H1N1 virus and the few changes of host transcription. Either the reduced virus replication efficiency of H1N1 virus is responsible for the lower host response. This is supported by previous study where the replication efficiency of the virus-cell system accounts for the level of the host innate immune response [49]. Or it is also possible that H1N1 viral replication is impaired because of its inability to modulate the host response, especially to induce proviral pathways. This hypothesis is based upon previous demonstration that stronger virus-induced MAPK activation resulted in higher viral replication efficiency [50].

Nevertheless, beyond these subtype-specific profiles, we were able to identify a list of 300 genes differentially expressed in both mock and infected samples. Strikingly, only about 5% of these genes were upregulated. A similar imbalance has previously been observed in other transcriptional profiles of infected cell lines [40], [51]. One could hypothesize that this may reflect the virally-induced cellular arrest of protein expression and could be due to the 5′cap snatching and subsequent degradation of cellular mRNA [52] and/or the inhibition of processing and export of cellular mRNA by NS1 [53]. Nevertheless these downregulated genes represented only 3.3% of the total number of genes detected, suggesting that a selective inhibition of their expression may occur during infection. The downregulated genes are implicated in different cellular processes such as ATP binding, regulation of translation, cellular protein complex assembly, glucose metabolic processes, cell cycle and apoptotic mitochondrial changes. On the other hand, the 16 genes found upregulated are specifically associated with innate cellular immunity. Seven of these are induced by interferon: *OAS1, ISG15, IRF7, OASL, ICAM1, IFITM1,* and *IFIT3.* These 7 ISGs have already been found upregulated together with other interferon genes upon H1N1 PR8 endothelial primary cell cultures infection [37] (Table S5). We also found an upregulation of *CFD*, a gene coding for a component of the alternative complement pathway. Complement is an important player in immunity and is induced by influenza infection [11], [54], [55]. Other induced genes of the infection signature determined in this study have never before been associated with influenza infection. They include *ETV3* which encodes a transcriptional repressor [56] that could be partially responsible for the downregulation of other genes belonging to the signature.

### Signature use for drug screening

Here we identified a list of genes whose expression is significantly altered during infection with different human and avian influenza virus subtypes. Since the outcome of infection appeared successful in our experimental conditions, it can be concluded that such a virally-induced cellular environment is favorable for virus replication. We therefore hypothesized that any molecule able to inverse the infection signature should be harmful to influenza virus replication. In contrast to many published transcriptomic studies [1], [11], we did not focus on a particular gene with a known function or large annotation that can be assumed to have a link with viral infection. To conduct the in silico screening, we filtered the infection signature genes according to their level of expression and selected the twenty most differentially expressed (with statistical significance) between mock and infected cells. We therefore took into account all of the information retrieved from the transcriptional analysis, which was a major advantage when using the Connectivity map. We selected eight molecules which induced gene expression modifications which anti-correlated with the infection signature. The hit-rate for this in silico screening was 0.53%.

Our experimental strategy presented several limitations: (i) we used a nylon microarray containing only 8000 genes thus meaning that the transcriptional profile of infected cells is incomplete; (ii) this profile was assessed for an established cell line, A549, which is different from those used in the Connectivity Map (MCF7, HL60 and PC3); (iii) the Connectivity Map contains data for only 1000 molecules and none of the molecules we identified was able to induce a full inversion of the infection signature (Figure 5C). Despite these limitations, seven molecules out of the eight selected by the in silico screening presented an antiviral effect on at least one of the tested viruses (87.5% of molecules confirmed). 2-aminobenzenesulfonamide and rilmenidine had only a modest antiviral effect on one specific virus (respectively H1N1 SOIV and H1N1 New Caledonia). Harmol and merbromin were weak inhibitors of most of the tested viruses. Brinzolamide and midodrine were weak to moderate inhibitors of most of the tested viruses. As expected, ribavirin was a strong inhibitor of all tested viruses. In light of these results, we conclude that we have identified a common signature whose partial inversion is strong enough to inhibit viral replication.

### Hypothesis on the mechanisms supporting a molecule's antiviral effect

We cannot rule out that some in silico selected drugs exert a possible direct effect on a viral activity or on a cellular pathway exploited by the virus. Among the seven molecules, three in particular could have such an effect: ribavirin and merbromin which could both directly inhibit a viral function, and harmol which could inhibit a proviral pathway. Harmol is a beta-carboline alkaloid of the medicinal plant, *Perganum harmala L*. (Zygophylaceae). Few specific effects are described for harmol except that it exerts a psychoactive effect by inhibiting monoamine oxydase [57], moderately inhibits platelet aggregation by inhibiting PLCγ2 [58] and induces apoptosis in some cell lines by activating caspase 8 [59]. PLCγ2 is implicated in the protein kinase C (PKC) activation pathway, the activity of which is crucial for influenza virus entry [60]. Therefore its inhibition by harmol could in part be responsible for the antiviral effect shown by this molecule. Likewise, activation of apoptosis could limit viral replication [61].

However, three types of evidence support our hypothesis that the selected molecules have an antiviral effect by modifying the host cell gene expression. First, the results of our test of infection efficiencies demonstrate that none of the molecules except for merbromin had an effect on viral structure or function before infection (Figure 6). Second, the high confirmation rate of the in silico selected drug panel validate the rational of the selection. Last, some molecules that regulated the host cell transcription in the same way that influenza virus infection enhanced viral production.

To our knowledge, modulation of the cell gene expression has never been described to support the effects of the in silico selected drug, except for ribavirin. This antiviral drug with in vitro activity against both DNA and RNA viruses [62], has several mechanisms of action proposed to support its antiviral effect (reviewed in [63]: i) the depletion of the intracellular GTP-pool by inhibition of inosine monophosphate dehydrogenase compromises the synthesis of progeny viral RNA; ii) the inhibition of viral RNA-dependent RNA polymerase activity has been shown for hepatitis C and influenza viruses; and iii) it could act as a RNA virus mutagen causing error catastrophe). Which mechanisms contribute to its anti-influenza effect in vivo remains undetermined. In this study, we selected ribavirin because it inversed the gene expression signature of infection, which could highlight a new potential antiviral mechanism of this molecule. An effect of ribavirin on the cellular gene expression has been reported to contribute to its antiviral effect on the respiratory syncytial virus (RSV) [64] and the hepatitis C virus (HCV) [65]. In these studies, ribavirin enhanced the expression of *ISG* in infected cells. It was concluded that ribavirin potentiates the interferon response induced by peginterferon (during treatment of HCV) [65] or induced by RSV infection [64]. However, ribavirin has also been shown to alter the expression of many genes implicated in various other cellular pathways such as apoptosis [66], cell cycle control or intracellular signaling [64]. We propose that these modifications contribute to its antiviral effect.

### Does this study now allow us to define co-factors and antiviral proteins?

None of the selected molecules fully inversed the infection signature. Therefore to try to identify anti or proviral factors, we first searched for genes whose expression could be inverted by all effective molecules. This was the case for only one gene, *calpain 1*, which was up-regulated by all the selected molecules and down-regulated during infection. The calpains, or calcium-regulated non-lysosomal thiol-proteases, are ubiquitous enzymes which catalyze limited proteolysis of substrates involved in cytoskeletal remodeling and signal transduction. We found no data in the literature describing any antiviral role for calpain 1. Such potential activity remains to be tested in the future.

It is also possible that each different molecule exerts its antiviral effect through different mechanisms and different combinations of gene expression modifications could be implied. These changes are listed in the Connectivity Map but except for midodrine and ribavirin, have yet to be confirmed by other studies. Midodrine is the prodrug of desglymidodrine, which is an alpha1-adrenergic receptor agonist used in the clinical management of patients with orthostatic hypotension [67]. Its effect on cellular gene expression can be derived from several microarray studies [68], [69] showing many transcriptional changes after stimulation of the alpha1 adrenoreceptor, involving for example genes encoding integrin-mediated cell adhesion proteins and proteins involved in hyaluronan signaling [70]. These observations are consistent with the observed midodrine-induced downregulation of *ICAM1* and *HYAL4* reported in the Connectivity Map. Both of these genes were up-regulated during infection. Their potential role in the influenza cell cycle remains to be determined.

Recently, several human RNAi screens identified host cell factors which are required for influenza virus replication [37], [71], [72], [73]. We wondered if the 20 genes of the concise infection signature were found to be important for the influenza virus in any of these screens (Table S5). Notably, the concise infection signature is specifically more enriched in regulators of influenza infection than random chance (compared to 8676 genes of the array, p-value = 0.0072 with Fisher's exact test). Four genes (*SLC2A2*, *ICAM1*, *OAS1*, *ISG15*) out of the 12 up-regulated genes were defined as proviral factors in these screens [37], [71], [72]. Three genes are ISGs: *ICAM1*, *OAS1* and *ISG15* that may be co-opted by the virus. Their down-regulation by the drugs could support partially their antiviral activity. On the other hand, none antiviral factor was identified in the list of 8 genes down-regulated during infection. This could be due to the low number of antiviral factors found by published screens (180 in total [37], [71], compared to 875 (unique) proviral factors [37], [71], [72], [73]). Therefore, the down-regulated genes of the infection signature can be considered as potential antiviral factors, which should be further tested.

### Outcomes and perspectives

To conclude, our investigation of transcriptional profiles of cells infected with different strains of influenza A viruses highlights virus specificity but, above all, has allowed us to define a universal influenza A virus-induced gene expression signature. Here we proposed to correlate this signature to gene expression profiles of cells treated by different molecules. This is the first study using the Connectivity Map to identify antivirals thought to act at the genomic level. One considerable advantage of some of these antivirals is their potential broad spectrum of action against all influenza A viruses, including novel pandemic viruses such as the H1N1 SOIV.

Except for harmol, all the antiviral molecules tested in this assay are approved for various different therapeutic indications. Our drug repositioning strategy should therefore contribute to the discovery of new alternative antivirals with accelerated regulatory registration. In the event of an unknown emerging virus, this approach may be of great interest to relatively quickly identify all available commercialized drugs with potential antiviral effects.

This study conducted in vitro in an established human cell line and with a nylon microarray represents a first proof of principle study. To identify effective anti-influenza molecules for use in clinical practice, we will now study the transcriptional response of patients to infection using a pan-genome microarray. Gene response to infection within a tissue in vivo should be more complex, with many cell types being implicated and with those infected being influenced by cytokines and the surrounding tissue.

Importantly, our dual experimental approach associating transcriptional study and in silico screening could be transferred to other pathogens. We are interested in identifying common gene-expression signatures of different viruses causing the same clinical disease to find useful therapies before etiologic diagnosis.

## Supporting Information

Figure S1 Boxplots for the 7 interferon stimulated genes (ISGs) upregulated during infection. These boxplots show the averaged normalized expression values of the 7 ISGs in each group of samples. The bottom edge of the box represents the 25th percentile of the data while the top edge of the boxplot represents the 75th percentile. The line inside the box represents the 50th percentile of the data or the median.
(0.13 MB TIF)

Figure S2 Neuraminidase assay is suitable for evaluation of the drug panel. A. Neuraminidase activity can be used to quantify virus. Different influenza viral stocks (A/New Caledonia/20/99 (H1N1), A/Moscow/10/99 (H3N2), A/Lyon/969/09 (H1N1 SOIV), A/Turkey/582/2006 (H5N1), A/Finch/England/2051/94 (H5N2), and A/Chicken/Italy/2076/99 (H7N1)) were serially diluted in DMEM. 25 µl of the viral dilutions were incubated with 75 µL of 20 µM MU-NANA for 1 hour at 37°C. Stop solution was added before reading the fluorescence. The signal to background ratio at each TCID50 is shown. Under these conditions, the signal to background ratio was proportional to the amount of virus between 2 and 70. At hightest titer, the enzyme activity reaches a plateau due to limiting substrate. Triton X-100 treatment of H5N1 viral stock enhanced NA activities, as previously described [27]. B. Molecules did not inhibit the neuraminidase activity of the influenza virus. A/Moscow/10/99 (H3N2) viral stock diluted in DMEM (107,8 TICD50/mL final) was incubated with increasing concentration of molecule (or DMEM for controls) for 0.5 h at room temperature before testing the neuraminidase activity as described in materials and methods.
(0.52 MB TIF)

Figure S3 Effective molecules inhibit H3N2 viral growth at early and later times of infection. A. A549 cells were treated with increasing concentrations of the molecule or solvent for 6 h and were subsequently infected with A/Moscow/10/99 (H3N2) influenza virus at a moi of 2. A neuraminidase test was performed at 24, 42 and 65 hpi to assess influenza viral growth. Values represent the mean of two independent experiments performed in duplicate. B. Potency of the inhibitors against A/Moscow/10/99 (H3N2) according to different times of infection. CC50: molecule concentration of 50% cytotoxicity; EC50: molecule concentration of 50% inhibition of viral replication; SI: selective index.
(0.89 MB TIF)

Figure S4 Concentration-viability curves of the eight molecules. A549 cells were treated with increasing concentrations of each molecule or solvent for 72 h and their viability was measured using Neutral Red dye (as described in materials and methods). Data are presented as a ratio of absorbance at 550 nm of treated cells to control cells. Values represent the mean of six independent experiments performed in duplicate, and error bars show the standard deviation of the mean. (+/− standard deviation). Horizontal lines are drawn to show the scatter of the control values (mean +/− standard deviation).
(0.34 MB TIF)

Figure S5 Six molecules inhibited H3N2 influenza viral growth. A549 cells were treated with increasing concentrations of the molecule or solvent for 6 h and were subsequently infected with A/Moscow/10/99 (H3N2) influenza A virus at a moi of 0.2 (Panel A) or 2 (Panel B). Viral titers were determined at 65 hpi using a neuraminidase test as described in materials and methods. Results in A and B are representative of three independent determinations in duplicate. Data are presented as a ratio of RFU (relative fluorescence unit) in supernatants of treated cells to RFU in control cells (mean +/− standard deviation). The dose-response curves are the results of a fit with a 3-parameter logistic equation (if at least one normalized response was less than 0.5). Horizontal lines are drawn to show the scatter of the control values (mean +/− standard deviation).
(0.68 MB TIF)

Figure S6 Concentration-viability curves of the seven positively correlated molecules. A549 cells were treated with increasing concentrations of each molecule or solvent for 72 h and their viability was measured using Neutral Red dye (as described in materials and methods). Data are presented as a ratio of absorbance at 550 nm of treated cells to control cells. Values represent the mean of three independent experiments performed in duplicate, and error bars show the standard deviation of the mean. (+/− standard deviation). Horizontal lines are drawn to show the scatter of the control values (mean +/− standard deviation).
(0.30 MB TIF)

Figure S7 Three molecules enhanced H3N2 influenza viral growth at a moi of 0.2. A549 cells were treated with increasing concentrations of the molecule or solvent for 6 h and were subsequently infected with A/Moscow/10/99 (H3N2) influenza A virus at a moi of 0.2 (Panel A) or 2 (Panel B). Viral titers were determined at 65 hpi using a neuraminidase test as described in materials and methods. Results in A and B are representative of two independent determinations in duplicate. Data are presented as a ratio of RFU (relative fluorescence unit) in supernatants of treated cells to RFU in control cells (mean +/− standard deviation). The dose-response curves are the results of a fit with a 3-parameter logistic equation (if at least one normalized response was less than 0.5). Horizontal lines are drawn to show the scatter of the control values (mean +/− standard deviation). Enhancement of H3N2 virus replication was verified by measuring viral titers at 65 hpi by end point titration assays in MDCK cells (TCID50/mL). For these assays, sample supernatants of duplicate were harvested at 65 hpi and stored at −80°C until analysis. Increase of viral titers were statistically significant for alvespimycin, methylbenzethoniumchloride, and sulodictil 40 µM (p-value <0.05, two tailed Welch t-test).
(0.64 MB TIF)

Figure S8 Five molecules inhibit H1N1 SOIV influenza viral growth. A549 cells were treated with increasing concentrations of the molecule or solvent for 6 h and were subsequently infected with A/Lyon/969/09 (H1N1 SOIV) influenza virus at a moi of 0.2 (Panel A) or 2 (Panel B). Viral titers were determined at 65 hpi using a neuraminidase test as described in materials and methods. This assay was performed in duplicate. Data are presented as the ratio of RFU in the supernatant of treated cells to RFU in control cells (mean +/− standard deviation). The dose-response curves are the results of a fit with 3-parameter logistic equation (if at least one normalized response was less than 0.5). Horizontal lines are drawn to show the scatter of the control values (mean +/− standard deviation).
(0.69 MB TIF)

Figure S9 Comparison of viral replication kinetics between different influenza A viruses in A549 cells. A549 cells were infected at a moi of 0.1 (Panel A) or at a moi of 2 (Panel B) with influenza virus A/New Caledonia/20/99 (H1N1), A/Lyon/969/09 (H1N1 SOIV), A/Moscow/10/99 (H3N2), A/Turkey/582/2006 (H5N1), A/Finch/England/2051/94 (H5N2), and A/Chicken/Italy/2076/99 (H7N1).
(0.45 MB TIF)

Table S1 Specific primers used for real-time quantitative RT-PCR.
(0.01 MB XLS)

Table S2 Gene expression data for 300 influenza virus regulated genes identified in our experiments. Values are log2 normalized gene expression intensities (see Experimental Procedures).
(0.28 MB XLS)

Table S3 Approved indications of the eight potential antivirals.
(0.01 MB XLS)

Table S4 Potency of the positively correlated drugs against A/Moscow/10/99 (H3N2). CC50: molecule concentration of 50% cytotoxicity; EC50: molecule concentration of 50% inhibition of viral replication; SI: selective index.
(0.01 MB XLS)

Table S5 Comparison between the 300 regulated genes in our experiment and identified factors required for influenza replication in published human RNAi screens. Genes belonging to the concise signature used to query connectivity map in our study are highlighted in grey. Negative regulators of influenza replication (antiviral) are genes whose depletion enhance viral infection. Positive regulators (proviral) knock-out decrease viral infection. Antiviral factors were identified in Shapira et al and Brass et al studies.
(0.03 MB XLS)
