# Supplementary figures and images for: Gene Expression Signature-Based Screening Identifies New Broadly Effective Influenza A Antivirals
Source: PLoS One. 2010 Oct 4;5(10):e13169. doi: 10.1371/journal.pone.0013169 (PMC2949399; doi:10.1371/journal.pone.0013169)

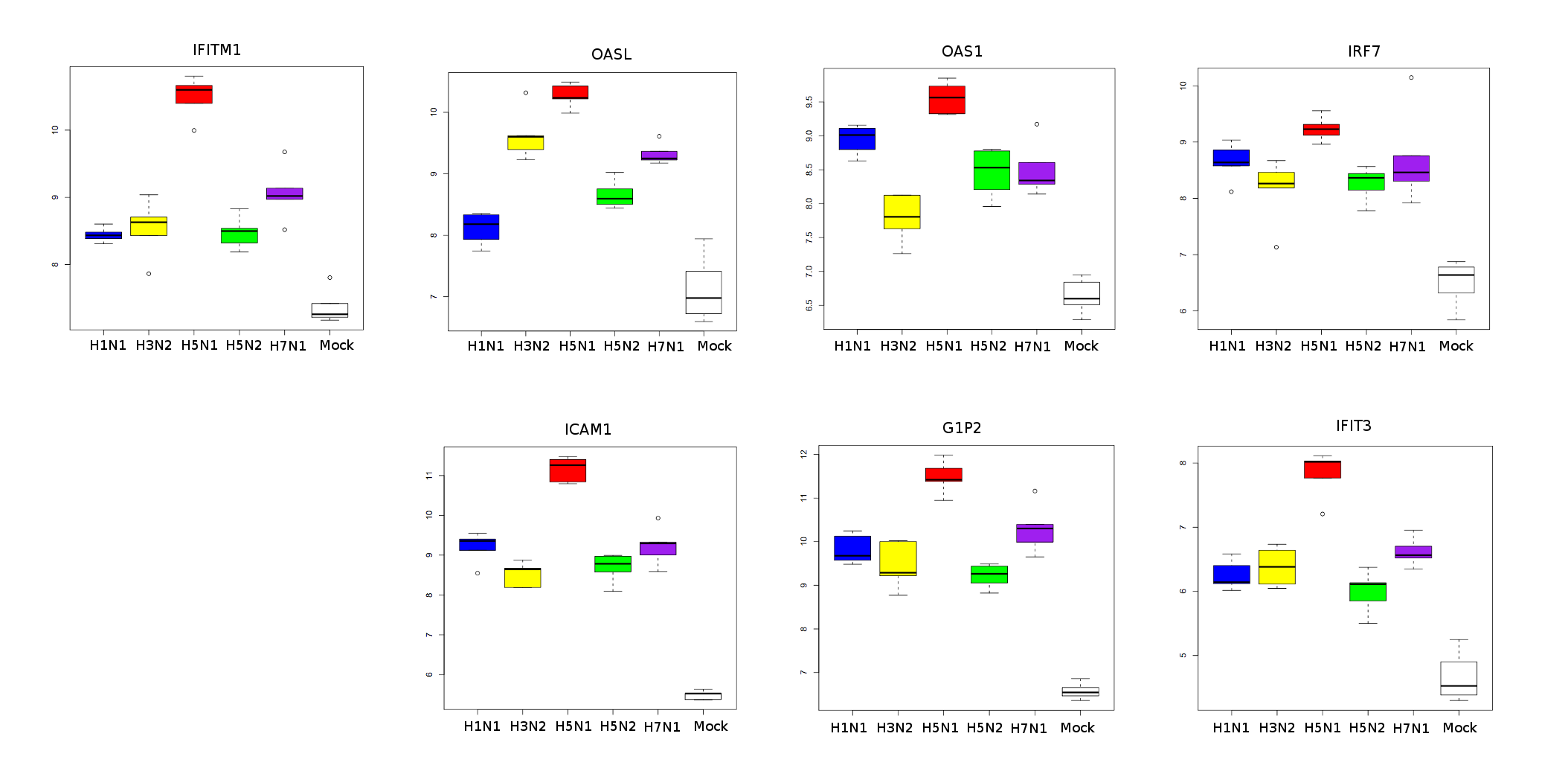

Supplement: Figure S1 — Boxplots for the 7 interferon stimulated genes (ISGs) upregulated during infection. These boxplots show the averaged normalized expression values of the 7 ISGs in each group of samples. The bottom edge of the box represents the 25th percentile of the data while the top edge of the boxplot represents the 75th percentile. The line inside the box represents the 50th percentile of the data or the median. (0.13 MB TIF) [file pone.0013169.s001.tif]

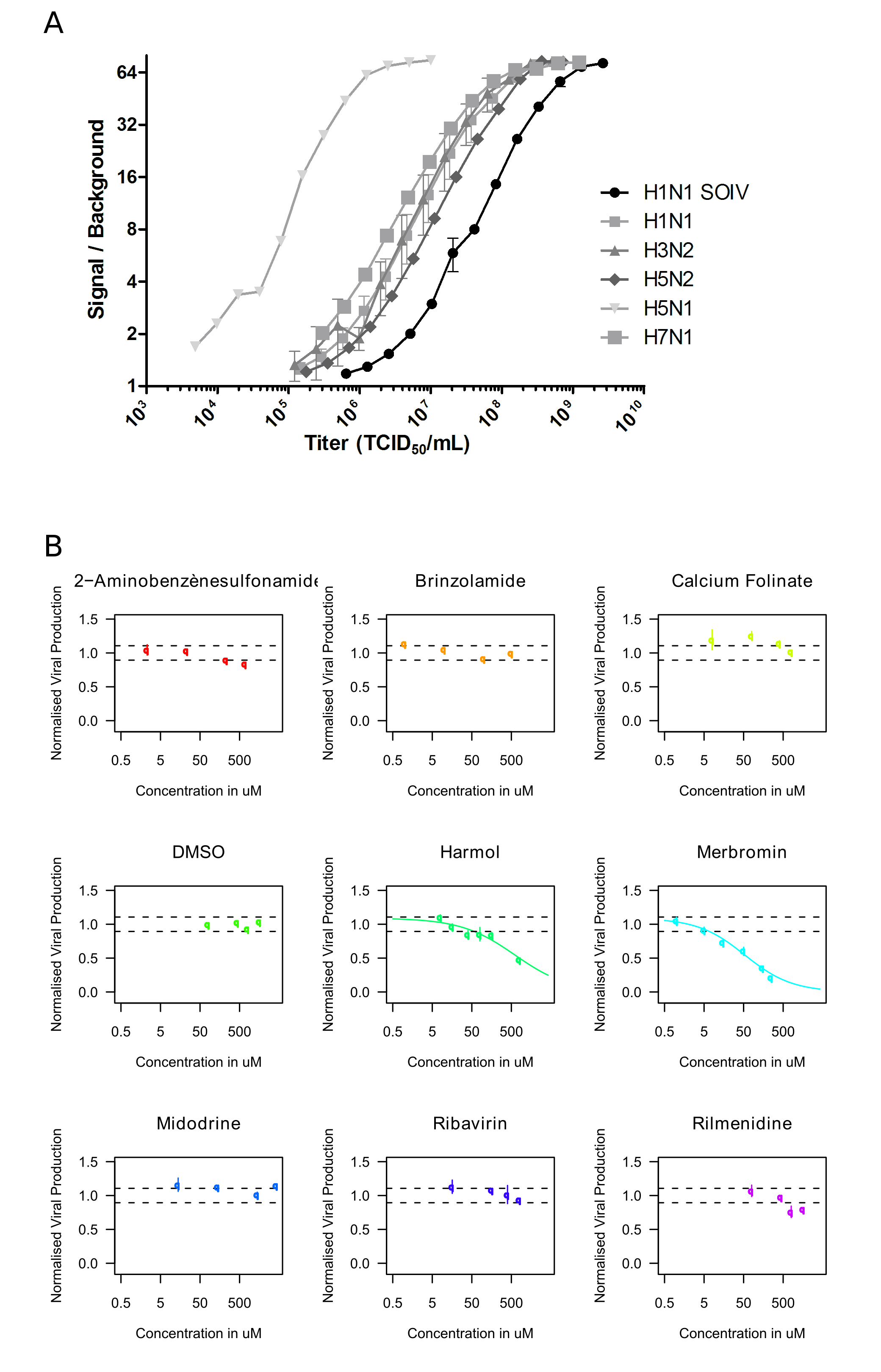

Supplement: Figure S2 — Neuraminidase assay is suitable for evaluation of the drug panel. A. Neuraminidase activity can be used to quantify virus. Different influenza viral stocks (A/New Caledonia/20/99 (H1N1), A/Moscow/10/99 (H3N2), A/Lyon/969/09 (H1N1 SOIV), A/Turkey/582/2006 (H5N1), A/Finch/England/2051/94 (H5N2), and A/Chicken/Italy/2076/99 (H7N1)) were serially diluted in DMEM. 25 µl of the viral dilutions were incubated with 75 µL of 20 µM MU-NANA for 1 hour at 37°C. Stop solution was added before reading the fluorescence. The signal to background ratio at each TCID50 is shown. Under these conditions, the signal to background ratio was proportional to the amount of virus between 2 and 70. At hightest titer, the enzyme activity reaches a plateau due to limiting substrate. Triton X-100 treatment of H5N1 viral stock enhanced NA activities, as previously described [27]. B. Molecules did not inhibit the neuraminidase activity of the influenza virus. A/Moscow/10/99 (H3N2) viral stock diluted in DMEM (107,8 TICD50/mL final) was incubated with increasing concentration of molecule (or DMEM for controls) for 0.5 h at room temperature before testing the neuraminidase activity as described in materials and methods. (0.52 MB TIF) [file pone.0013169.s002.tif]

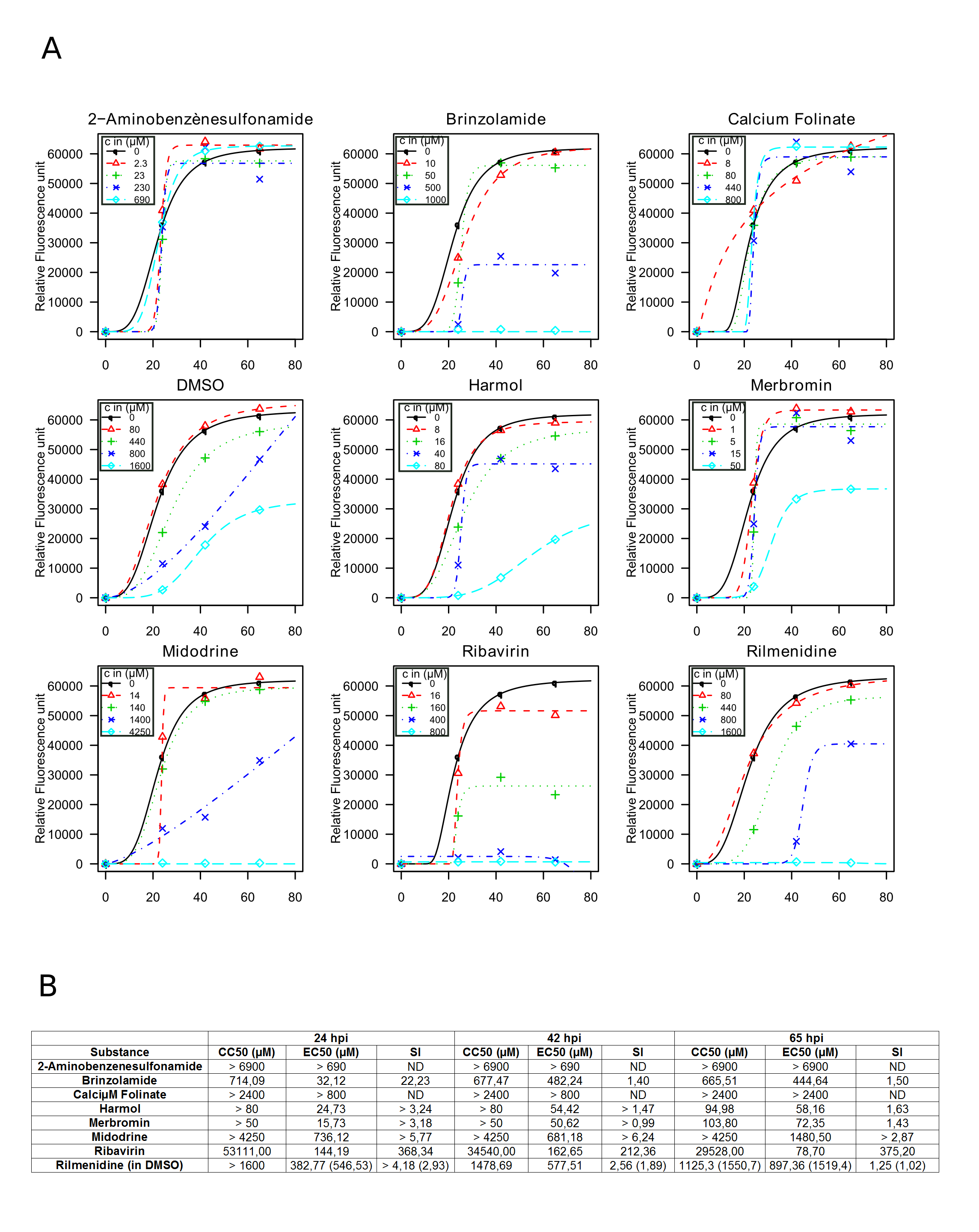

Supplement: Figure S3 — Effective molecules inhibit H3N2 viral growth at early and later times of infection. A. A549 cells were treated with increasing concentrations of the molecule or solvent for 6 h and were subsequently infected with A/Moscow/10/99 (H3N2) influenza virus at a moi of 2. A neuraminidase test was performed at 24, 42 and 65 hpi to assess influenza viral growth. Values represent the mean of two independent experiments performed in duplicate. B. Potency of the inhibitors against A/Moscow/10/99 (H3N2) according to different times of infection. CC50: molecule concentration of 50% cytotoxicity; EC50: molecule concentration of 50% inhibition of viral replication; SI: selective index. (0.89 MB TIF) [file pone.0013169.s003.tif]

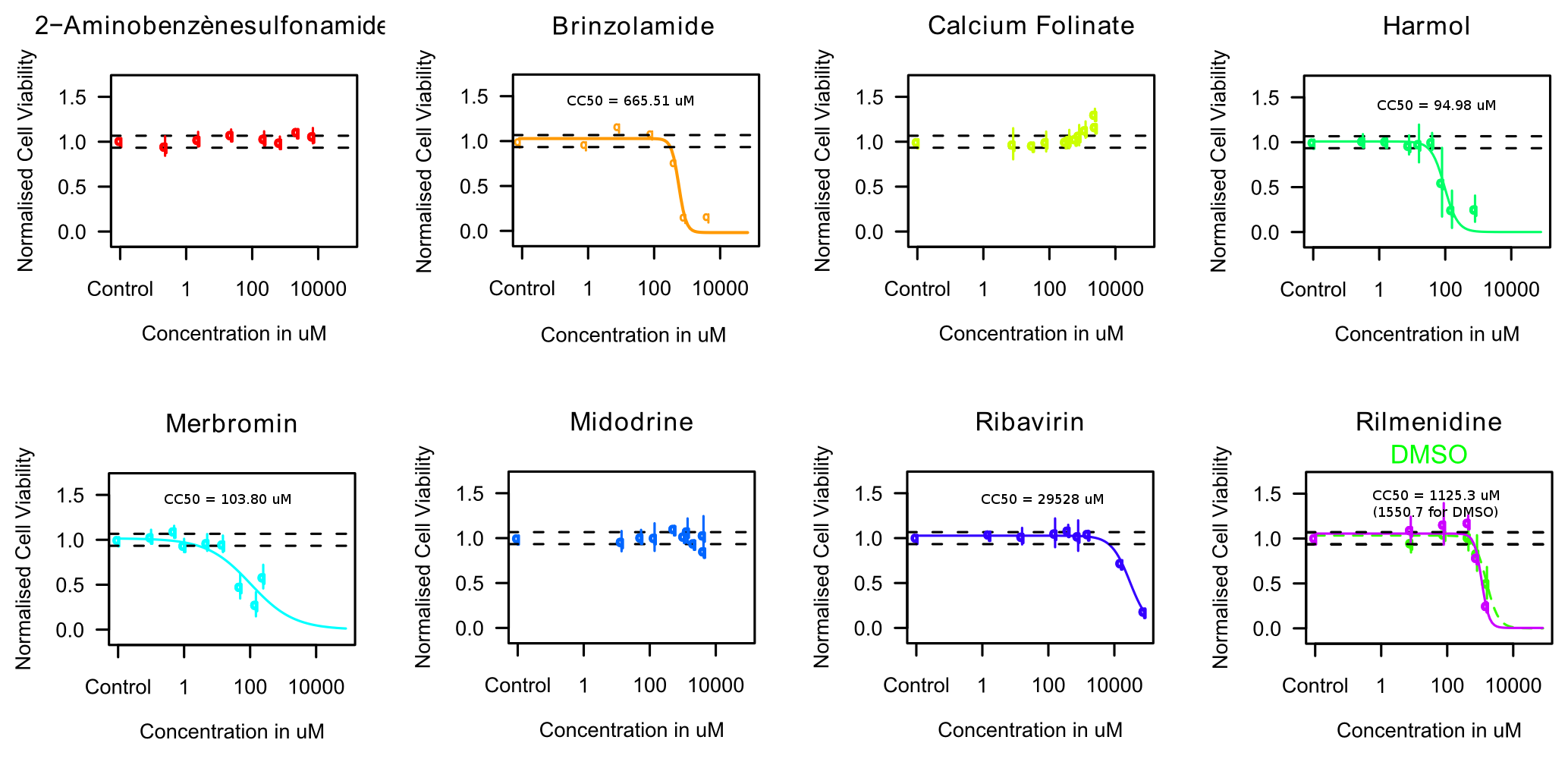

Supplement: Figure S4 — Concentration-viability curves of the eight molecules. A549 cells were treated with increasing concentrations of each molecule or solvent for 72 h and their viability was measured using Neutral Red dye (as described in materials and methods). Data are presented as a ratio of absorbance at 550 nm of treated cells to control cells. Values represent the mean of six independent experiments performed in duplicate, and error bars show the standard deviation of the mean. (+/− standard deviation). Horizontal lines are drawn to show the scatter of the control values (mean +/− standard deviation). (0.34 MB TIF) [file pone.0013169.s004.tif]

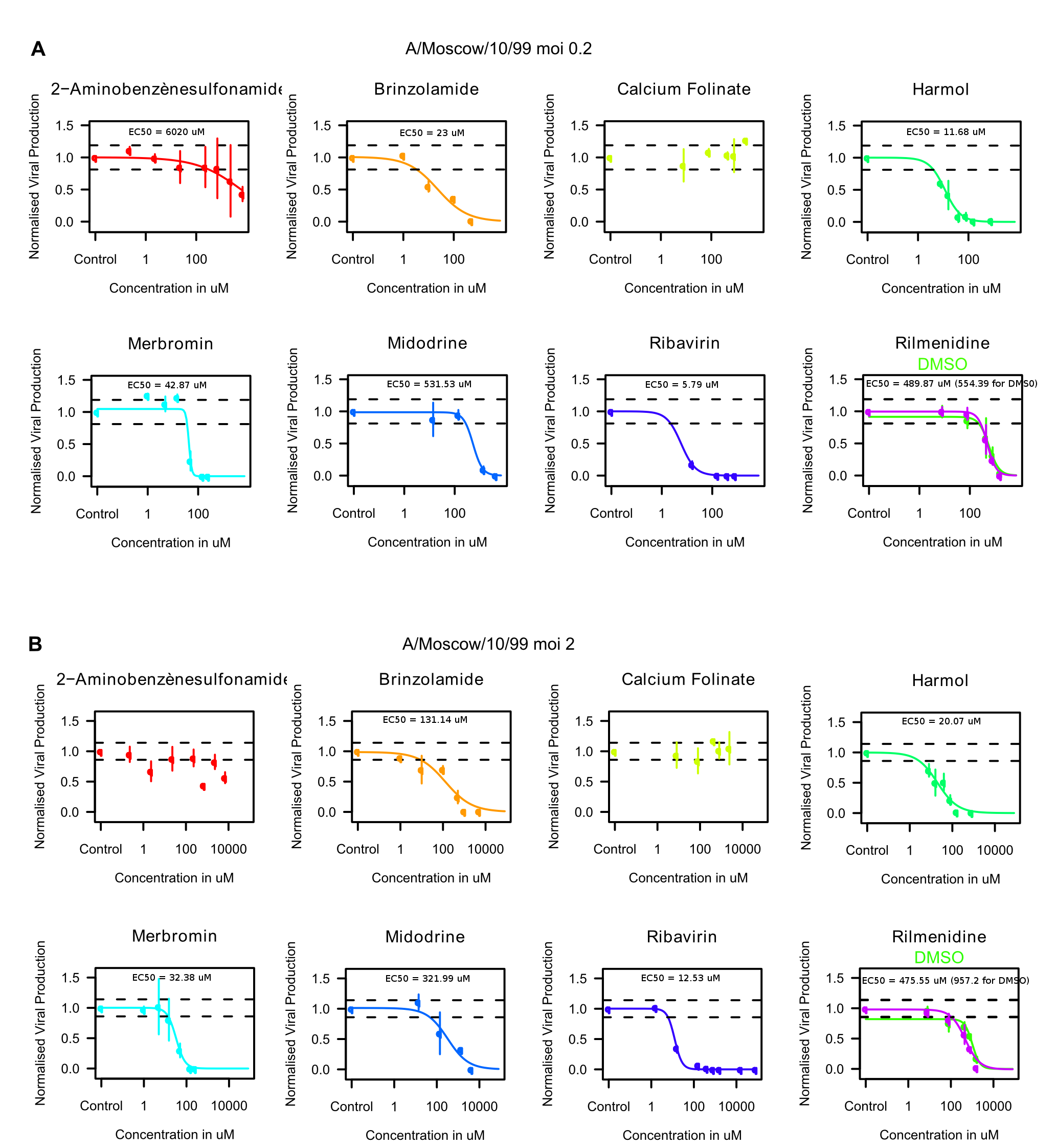

Supplement: Figure S5 — Six molecules inhibited H3N2 influenza viral growth. A549 cells were treated with increasing concentrations of the molecule or solvent for 6 h and were subsequently infected with A/Moscow/10/99 (H3N2) influenza A virus at a moi of 0.2 (Panel A) or 2 (Panel B). Viral titers were determined at 65 hpi using a neuraminidase test as described in materials and methods. Results in A and B are representative of three independent determinations in duplicate. Data are presented as a ratio of RFU (relative fluorescence unit) in supernatants of treated cells to RFU in control cells (mean +/− standard deviation). The dose-response curves are the results of a fit with a 3-parameter logistic equation (if at least one normalized response was less than 0.5). Horizontal lines are drawn to show the scatter of the control values (mean +/− standard deviation). (0.68 MB TIF) [file pone.0013169.s005.tif]

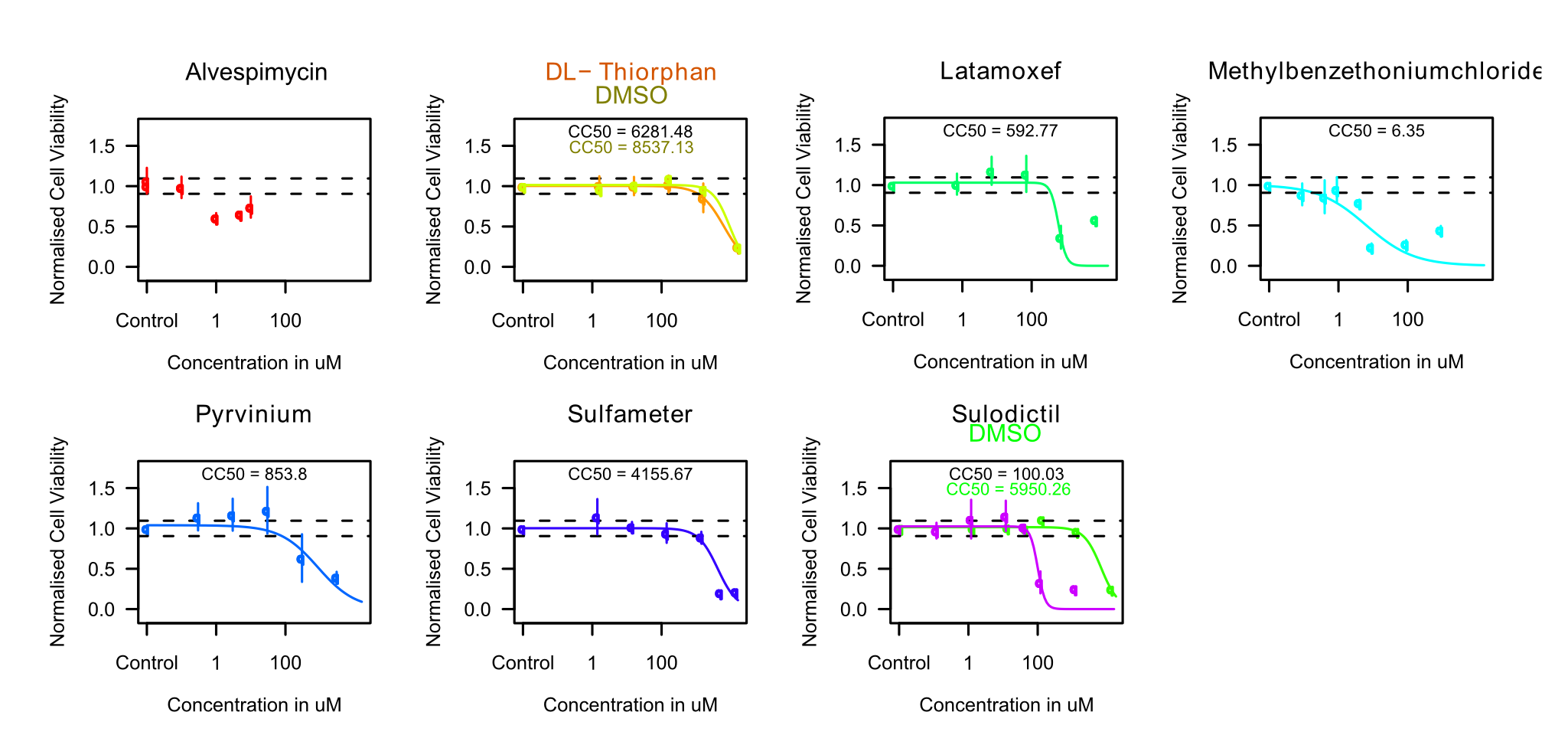

Supplement: Figure S6 — Concentration-viability curves of the seven positively correlated molecules. A549 cells were treated with increasing concentrations of each molecule or solvent for 72 h and their viability was measured using Neutral Red dye (as described in materials and methods). Data are presented as a ratio of absorbance at 550 nm of treated cells to control cells. Values represent the mean of three independent experiments performed in duplicate, and error bars show the standard deviation of the mean. (+/− standard deviation). Horizontal lines are drawn to show the scatter of the control values (mean +/− standard deviation). (0.30 MB TIF) [file pone.0013169.s006.tif]

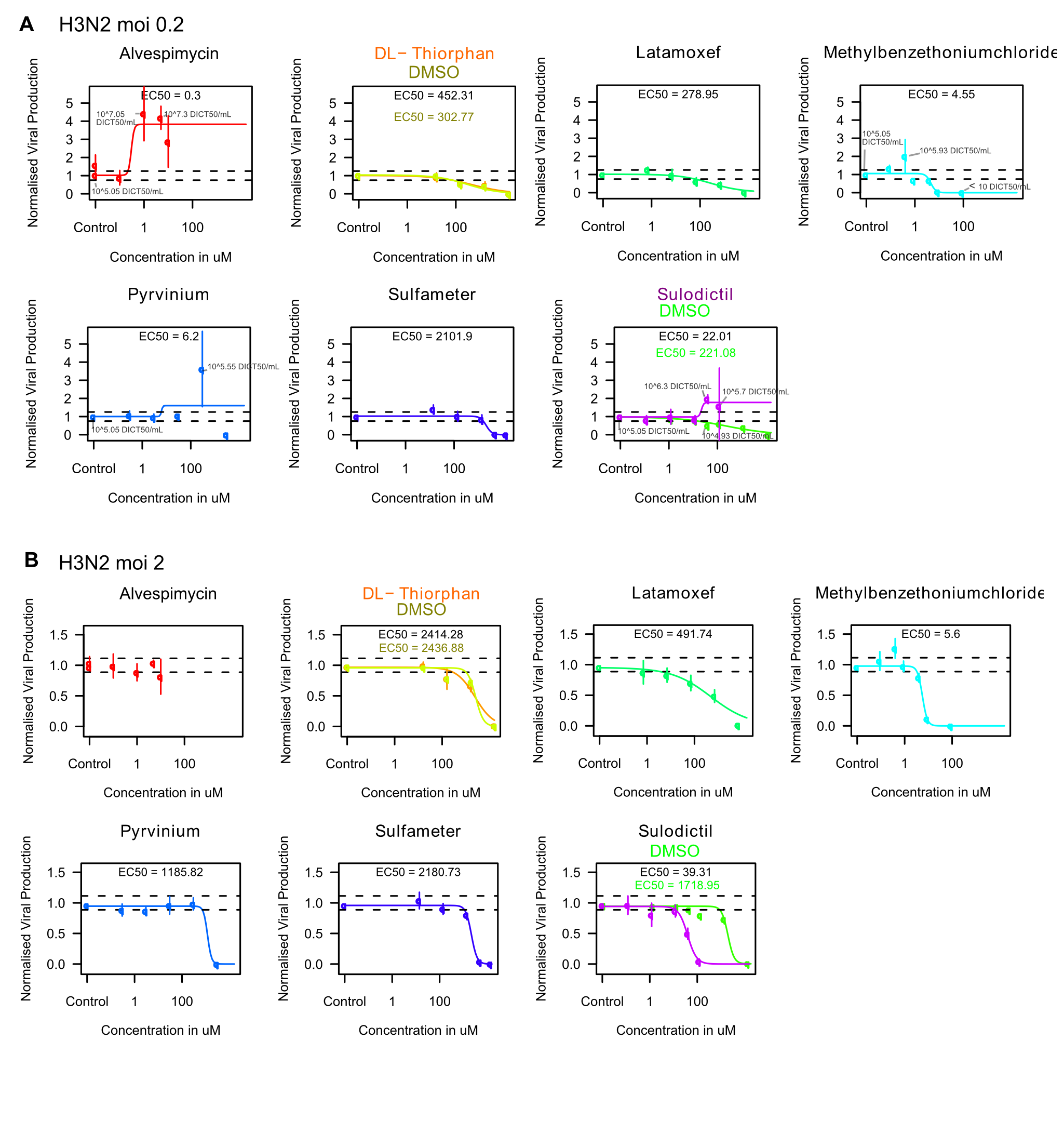

Supplement: Figure S7 — Three molecules enhanced H3N2 influenza viral growth at a moi of 0.2. A549 cells were treated with increasing concentrations of the molecule or solvent for 6 h and were subsequently infected with A/Moscow/10/99 (H3N2) influenza A virus at a moi of 0.2 (Panel A) or 2 (Panel B). Viral titers were determined at 65 hpi using a neuraminidase test as described in materials and methods. Results in A and B are representative of two independent determinations in duplicate. Data are presented as a ratio of RFU (relative fluorescence unit) in supernatants of treated cells to RFU in control cells (mean +/− standard deviation). The dose-response curves are the results of a fit with a 3-parameter logistic equation (if at least one normalized response was less than 0.5). Horizontal lines are drawn to show the scatter of the control values (mean +/− standard deviation). Enhancement of H3N2 virus replication was verified by measuring viral titers at 65 hpi by end point titration assays in MDCK cells (TCID50/mL). For these assays, sample supernatants of duplicate were harvested at 65 hpi and stored at −80°C until analysis. Increase of viral titers were statistically significant for alvespimycin, methylbenzethoniumchloride, and sulodictil 40 µM (p-value <0.05, two tailed Welch t-test). (0.64 MB TIF) [file pone.0013169.s007.tif]

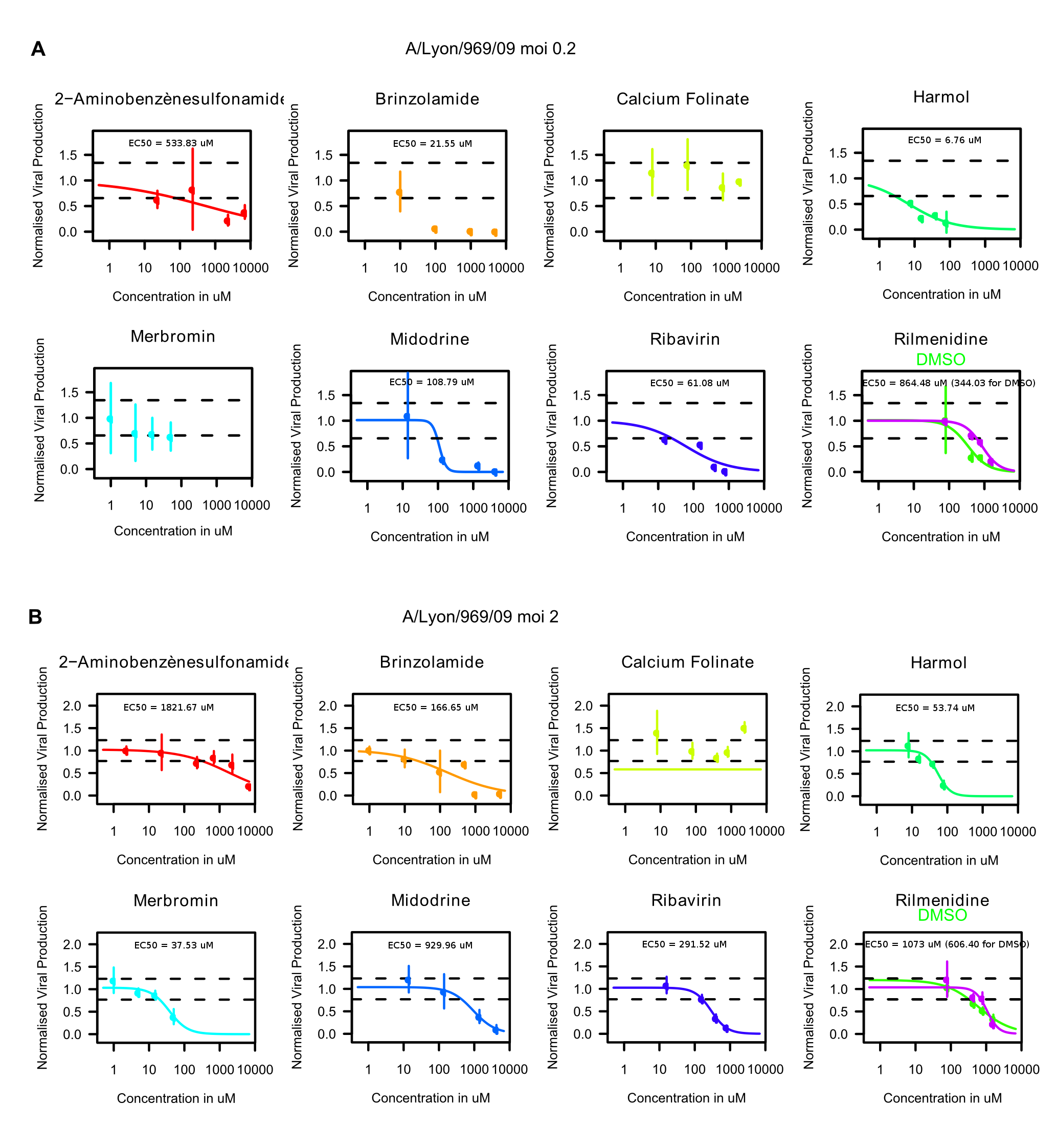

Supplement: Figure S8 — Five molecules inhibit H1N1 SOIV influenza viral growth. A549 cells were treated with increasing concentrations of the molecule or solvent for 6 h and were subsequently infected with A/Lyon/969/09 (H1N1 SOIV) influenza virus at a moi of 0.2 (Panel A) or 2 (Panel B). Viral titers were determined at 65 hpi using a neuraminidase test as described in materials and methods. This assay was performed in duplicate. Data are presented as the ratio of RFU in the supernatant of treated cells to RFU in control cells (mean +/− standard deviation). The dose-response curves are the results of a fit with 3-parameter logistic equation (if at least one normalized response was less than 0.5). Horizontal lines are drawn to show the scatter of the control values (mean +/− standard deviation). (0.69 MB TIF) [file pone.0013169.s008.tif]

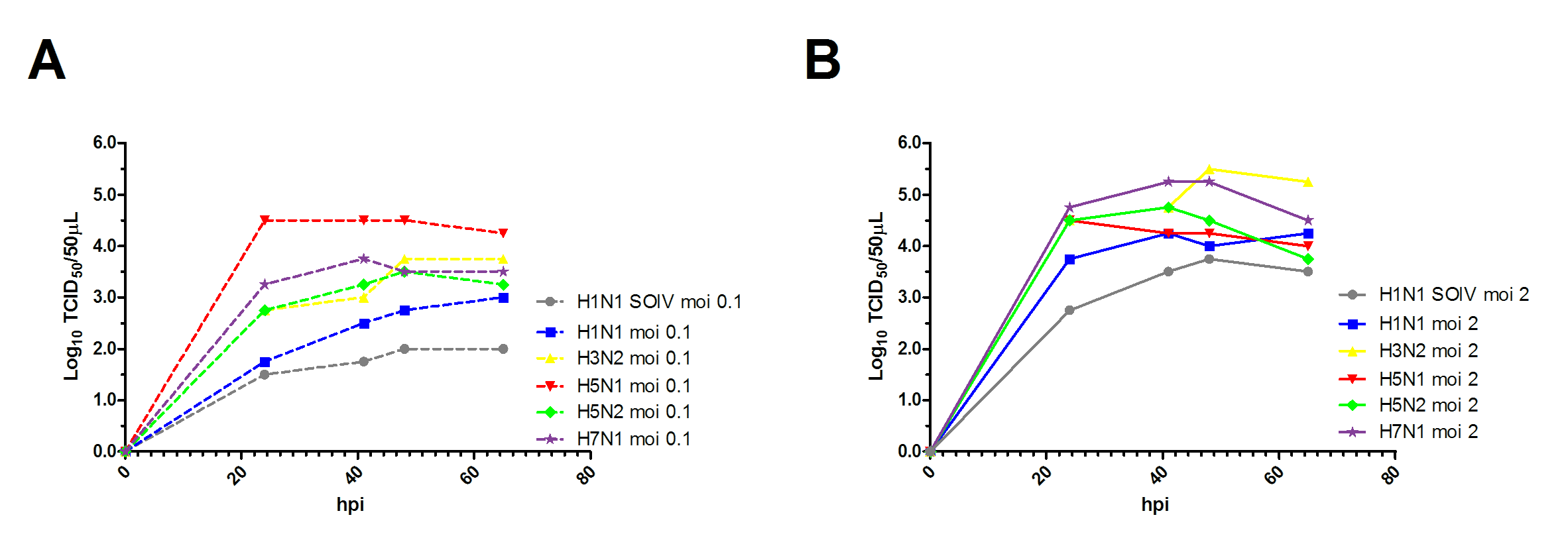

Supplement: Figure S9 — Comparison of viral replication kinetics between different influenza A viruses in A549 cells. A549 cells were infected at a moi of 0.1 (Panel A) or at a moi of 2 (Panel B) with influenza virus A/New Caledonia/20/99 (H1N1), A/Lyon/969/09 (H1N1 SOIV), A/Moscow/10/99 (H3N2), A/Turkey/582/2006 (H5N1), A/Finch/England/2051/94 (H5N2), and A/Chicken/Italy/2076/99 (H7N1). (0.45 MB TIF) [file pone.0013169.s009.tif]
